# A Guide to Enterotypes across the Human Body: Meta-Analysis of Microbial Community Structures in Human Microbiome Datasets

**DOI:** 10.1371/journal.pcbi.1002863

**Published:** 2013-01-10

**Authors:** Omry Koren, Dan Knights, Antonio Gonzalez, Levi Waldron, Nicola Segata, Rob Knight, Curtis Huttenhower, Ruth E. Ley

**Affiliations:** 1 Department of Microbiology, Cornell University, Ithaca, New York, United States of America; 2 Department of Computer Science, University of Colorado, Boulder, Colorado, United States of America; 3 Department of Biostatistics, Harvard School of Public Health, Boston, Massachusetts, United States of America; 4 Department of Biostatistics and Computational Biology, Dana-Farber Cancer Institute, Boston, Massachusetts, United States of America; 5 Department of Chemistry and Biochemistry, University of Colorado, Boulder, Colorado, United States of America; 6 Howard Hughes Medical Institute, University of Colorado, Boulder, Colorado, United States of America; University of California Davis, United States of America

## Abstract

Recent analyses of human-associated bacterial diversity have categorized individuals into ‘enterotypes’ or clusters based on the abundances of key bacterial genera in the gut microbiota. There is a lack of consensus, however, on the analytical basis for enterotypes and on the interpretation of these results. We tested how the following factors influenced the detection of enterotypes: clustering methodology, distance metrics, OTU-picking approaches, sequencing depth, data type (whole genome shotgun (WGS) vs.16S rRNA gene sequence data), and 16S rRNA region. We included 16S rRNA gene sequences from the Human Microbiome Project (HMP) and from 16 additional studies and WGS sequences from the HMP and MetaHIT. In most body sites, we observed smooth abundance gradients of key genera without discrete clustering of samples. Some body habitats displayed bimodal (*e.g.*, gut) or multimodal (*e.g.*, vagina) distributions of sample abundances, but not all clustering methods and workflows accurately highlight such clusters. Because identifying enterotypes in datasets depends not only on the structure of the data but is also sensitive to the methods applied to identifying clustering strength, we recommend that multiple approaches be used and compared when testing for enterotypes.

## Author Summary

Recent work has suggested that individuals can be classified into ‘enterotypes’ based on the abundance of key bacterial taxa in gut microbial communities. However, the generality of enterotypes across populations, and the existence of similar cluster types for other body sites, remains to be evaluated. We combined the Human Microbiome Project 16S rRNA gene sequence data and metagenomes with similar published data to assess the existence of enterotypes across body sites. We found that rather than forming enterotypes (note we use this term for clusters in all body sites), most samples fell into gradients based on taxonomic abundances of bacteria such as *Bacteroides*, although in some body sites there is a bi/multi modal distribution of samples across gradients. Furthermore, many of the methods used in the analysis (*e.g.*, distance metrics and clustering approaches) affected the likelihood of identifying enterotypes in particular body habitats. We recommend that multiple approaches be used and compared when testing for enterotypes.

## Introduction

Together with the MetaHIT consortium [1], the Human Microbiome Project (HMP) represents one of the first major attempts to define the microbial diversity comprising the “normal healthy” human microbiome [2]. The HMP dataset includes 16S rRNA gene sequence data of roughly twice the size of all similarly derived data in previously published studies, effectively tripling the size of combined data available for comparative studies (Table S1). In addition, the HMP generated whole-genome shotgun (WGS) metagenomic data for a subset of individuals. These data allowed for the characterization of patterns of microbial diversity across body sites and between individuals [2].

The HMP data also provides an opportunity to test the generality of the concept of enterotypes in the human microbiome. Arumugam *et al.* first articulated the concept of enterotypes as robust clustering of human gut samples based on microbial community composition, and largely driven by the abundances of key bacterial genera [3]. Although the term ‘enterotype’ refers to microbiota types within the gut, the concept can be applied generally, and here, for convenience, we use the term ‘enterotype’ to refer to microbiota types across different body sites. The HMP data are ideally suited to test the robustness of the enterotype concept in multiple body sites, and together with recently published community-generated datasets, across multiple populations.

In this report, we combined 16S rRNA gene sequence data generated using next-generation sequencing by the scientific community (hereafter, ‘community data’) together with the MetaHIT WGS data [3] and the recently released HMP 16S rRNA gene sequence data and WGS data [2]. Because there is currently no community standard for testing for enterotypes, we explore how the detection of enterotypes is affected by the following: clustering methodology, distance metrics, OTU-picking approaches, sequencing depth (*i.e.*, rarefaction), data type (16S rRNA vs. WGS), and the specific region of the 16S rRNA gene sequenced. We find that the emergence of enterotypes is sensitive to the community structure of communities within each body site, and importantly also to the analysis methods employed. Our comparative analysis of various approaches across datasets informs the discussion on the technical basis for enterotyping and on how to interpret enterotype results.

## Materials and Methods

### 16S rRNA Gene Sequence Processing

We constructed a database containing the recently released HMP 16S rRNA gene sequence data [4] and publically available (published) human microbiome datasets (community data). For inclusion, community datasets were required to contain a minimum of 25 samples per study and sequences generated using the Roche 454 platform (Table S1). The majority of samples were from healthy controls; however, a small subset of samples was derived from subjects that differed from adult healthy subjects due to age (*i.e*., infants and the elderly), use of antibiotics, or possible presence of disease (Fig. S1). We acquired raw SFF files and metadata files containing the unique identifiers for each sample within a study (barcodes) from the authors and re-processed the data using the default settings in the Quantitative Insights Into Microbial Ecology (QIIME) analysis pipeline [5]. For the majority of samples, quality filtering consisted of rejecting reads <200 nt and >1000 nt, excluding homopolymer runs >6 nt, accepting 0 barcode corrections and 0 primer mismatches; two datasets were processed with slightly different screening parameters, as described in their respective publications [6], [7]. When picking operational taxonomic units (OTUs) we used the OTU tables generated by the HMP, which were created *de novo*. Because the regions of the 16S rRNA gene differed between studies (and within: the HMP sequenced both V1–V3 and V3–V5 regions), we used a reference-based approach (hence, we did not denoise the data) to pick OTUs at 97% pairwise identity using as a reference the latest release of the GreenGenes (GG) taxonomy [8]. We also used the phylogenetic tree from GG to calculate weighted (abundance based) and unweighted (presence/absence based) UniFrac distances between communities [9], after applying two rarefactions (1,000 and 2,000 sequences/sample) to standardize sequence counts. Principal coordinates analysis (PCoA) was applied to the distance matrices for visualization.

### Metagenomic Data Processing

The HMP and MetaHIT shotgun metagenomic datasets were taxonomically profiled using MetaPhlAn [10] (version 1.1, default parameter settings), which infers relative abundances for all taxonomic levels (from phyla to species) for Bacteria and Archaea. We performed standard quality control on the HMP and MetaHIT samples as reported in the original studies [2], [3] - other metagenomic pre-processing steps (*e.g.*, error detection, assembly, or gene annotation) are not required by MetaPhlAn. The taxonomic profiles of HMP metagenomes are available at http://www.hmpdacc.org/HMSMCP/, and the MetaHIT profiles can be downloaded from http://huttenhower.sph.harvard.edu/metaphlan/. The 690 HMP metagenomic samples from 7 different body sites can be accessed at http://hmpdacc.org/HMASM/, from which we used the ‘WGS’ reads (*i.e.*, we did not use the ‘PGA’ assemblies), collapsing multiple visits from the same individual into one sample. The 124 fecal samples from MetaHIT were downloaded from the European Nucleotide Archive (http://www.ebi.ac.uk/ena/, study accession number ERP000108).

### Enterotyping

To evaluate the clustering results in the context of previously published results reporting enterotypes, we merged publicly available data (genus relative abundance tables) from MetaHIT [3] with data for the 16S rRNA-based HMP and non-HMP samples, based on genus-level taxonomy assignments. We performed enterotype testing using the relative abundances of OTUs (rarified at 1,000 sequences/sample for the majority of analyses, except where effect of rarefaction was tested specifically), to which we applied five distance metrics: Jensen-Shannon divergence (JSD), Root Jensen-Shannon divergence (rJSD), Bray-Curtis (BC), and weighted/unweighted UniFrac distances. For the calculation of JSD and BC distances, we first binned the counts of OTUs at the desired level (95% and 97% ID for genus and species level OTUs, respectively). We used the R “vegan” package [11] for calculating the Bray-Curtis distance according to this formula for the distance between samples *j* and *k*, with taxa/OTUs indexed by *i*:
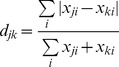
Clustering was performed via partitioning around medoids in the R package “cluster” [12]. We chose the number of clusters and quality of the resulting clusters by maximizing the prediction strength (PS) [13] and silhouette index (SI) [14]. We applied a criterion of ≥0.90 for PS to signify strong clustering (this implies that 90% of the data points fall within the cluster and 10% are outliers). For SI, we used a score of 0.5 for moderate clustering as described by Wu *et al.* [15], and ≥0.75 for strong clustering (note this is close to the value of 0.71 originally reported for strong clustering [16]). We performed kernel density estimation of the global distribution of gut microbial communities using the R package “ks” [17]. This included automatic inference of unconstrained (non-diagonal) bandwidth parameters using the function “Hscv”. We also calculated the Caliński-Harabasz (CH) statistic for comparison to PS and SI, using the R ‘fpc’ package [18]. This package uses the following formula for the CH statistic [19]:
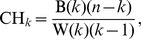
where
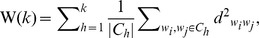
and
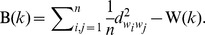
In this formula, *n* is the number of data points *w*, *k* is the number of clusters, and *C_h_* represents the set of data points in cluster *h*. We chose to use PS and SI to assess the support for clustering (and to choose the number of clusters if supported), as they are both absolute measures of the clustering quality, while CH is only a relative assessment of the quality of the clustering.

### Synthetic Dataset

We generated a synthetic dataset of 100 communities each containing 3,000 “sequences” belonging to 500 mock OTUs. For each synthetic community, 90% (2,700 sequences, or OTU observations) was drawn from the same randomly generated lognormal abundance distribution (shared across all communities) and the remaining 10% (300 sequences) drawn from one of four unique lognormal distributions, forcing the data into four clusters. We then applied the enterotyping methods as described above.

## Results and Discussion

### Mapping HMP Diversity onto Community-Generated Diversity

**Figure 1:**
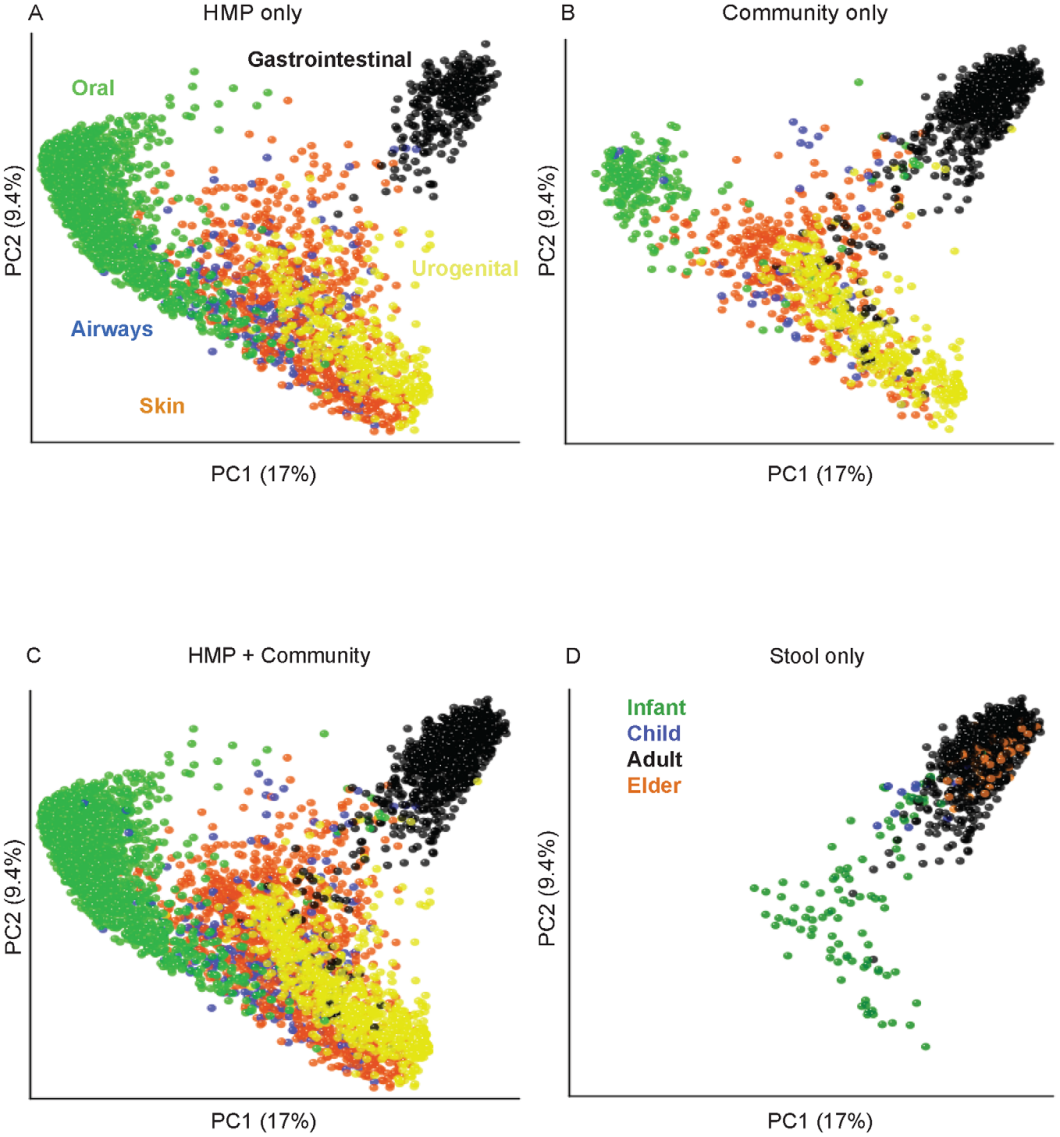
Bacterial diversity clusters by body habitat. A–C: All body sites. The two principal coordinates from the PCoA analysis of the unweighted UniFrac distances are plotted for (A) HMP data; (B) community data (see Table S1 for list of studies), (C) both datasets combined. Symbol colors correspond to body sites as indicated on panel A. Panel D shows gut samples (majority are fecal) divided into infants (green), children (blue), adults (black) and elderly (orange) samples. The variance explained by the PCs is indicated in parentheses on the axes.

Beta-diversity measures provide a view of how diversity differs between sets of samples and quantifies those differences. We used the unweighted UniFrac measure of β-diversity to contrast the range of bacterial phylogenetic diversity captured by the HMP data to existing community data (Fig. 1). This analysis showed that the overall pattern of diversity is similar for HMP and community data, with clear separation between body sites (Figs. 1A, B, S1) as has been described previously [2], [20]. Similarly, Fig. S2 shows the locations of the MetaHIT samples relative to the HMP and other community fecal samples. The HMP and MetaHIT data map onto the community data well (Fig. 1C; Fig. S2), lending support for the approach of combining these sets in a meta-analysis of body habitats.

The gut microbiota are the most extensively studied of the human-associated microbiota. Combining community and HMP fecal microbial 16S rRNA gene sequence data effectively extended the subject age range from early infancy to old age (3 days to 85 years old), with the HMP supplying the majority of the middle years of the human life span. Interestingly, infant samples (younger than 2.5 years) were outliers in the range of diversity represented by the healthy adult and elder (older than 70 years) gut (Fig. 1D) and were more similar to vaginal and skin communities. Adult HMP samples cluster together with those from the community studies, excluding samples from infants (<2.5 yrs) and elders (Table S1). This combined analysis corroborates the previously described vast difference between bacterial diversity of infants and adults [21], [22].

### Effect of Clustering Methodology

**Figure 2:**
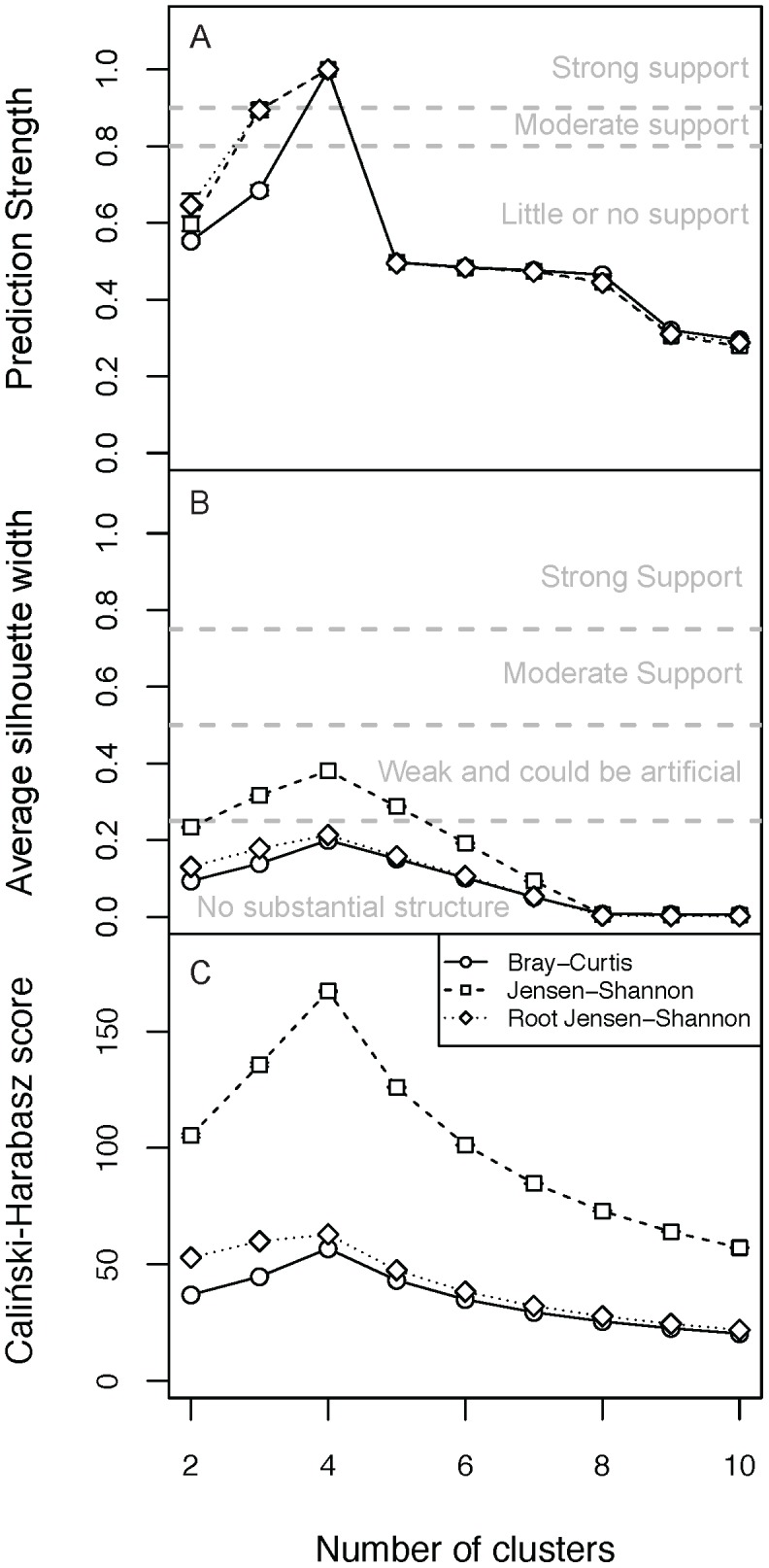
A positive control of cluster structure recovered from lognormally distributed synthetic community data containing four clusters. Presence of enterotypes was tested using: (A) prediction strength, (B) silhouette index and (C) Caliński-Harabasz combined with BC, JSD and rJSD distance metrics. Bars are standard errors.

We first tested the effects of different cluster scoring methods using a lognormally distributed synthetic community data containing 4 clusters that served as a positive control for enterotypes. We applied the JSD, rJSD and BC distance measures to the synthetic dataset and compared cluster scores using prediction strength (PS, Fig. 2A), silhouette index (SI, Fig. 2B) and Calińksi-Harabasz (CH, Fig. 2C) scores. This analysis revealed strong support for 4 clusters using PS for the BC, JSD and rJSD distance metrics, but SI provided no support for clustering using BC and rJSD, and only weak support for 3–5 clusters using the JSD distance metric. The CH index supported 4 clusters using only the JSD distance metric. Wu *et al.* also reported a discrepancy in cluster scoring strengths between clustering methods [15]: CH indicated that 3 enterotypes were present, but SI provided weak support using rJSD. Wu *et al.* also compared clustering with CH and SI together with weighted/normalized unweighted UniFrac, BC and Euclidean distances, and reported concordant numbers of clusters with weighted UniFrac only. Together these results indicate that these different clustering methodologies can yield inconsistent results, although SI and CH have been reported to be stable and comparable [23], [24].

Arumugan *et al.* used CH as the basis for choosing the number of enterotypes, even when SI values were very low (all published values were less than or equal to 0.25), indicating weak or no support for clustering [3]. It is important to note that the CH score is a relative measure that alone cannot be used to determine statistical significance of clustering in the data, and that furthermore, CH is intended to indicate the optimal number of clusters based on the assumption that clusters exist. PS and SI, on the other hand, are absolute measures of how likely cluster structure is to emerge from a dataset. Based on our results, we recommend using at least one absolute measure (specifically, we recommend PS), and if possible confirming those results with an additional absolute measure (such as SI), when searching for enterotypes. Depending on the signal-to-noise distribution within individual datasets and data types, PS may have difficulty identifying clusters represented by few samples, as we discuss below (*e.g.*, posterior fornix WGS data). In such cases SI may be relied on, but we recommend using a high threshold (*e.g.*, ≥0.75) in identifying potentially reproducible clusters. We prefer PS over SI for large sample sizes because (1) it has a clear quantitative and intuitive interpretation, (2) it allows estimation of the clustering stability of individual samples, and (3) it performs better than SI in recovering known enterotypes in synthetic datasets. Note however that there is currently no consensus in the field on the specific thresholds that should be used with these methods for assessing clustering strength, making it all the more important for researchers to clearly state the criteria they apply when reporting enterotypes.

### Effect of Distance Metric

**Figure 3:**
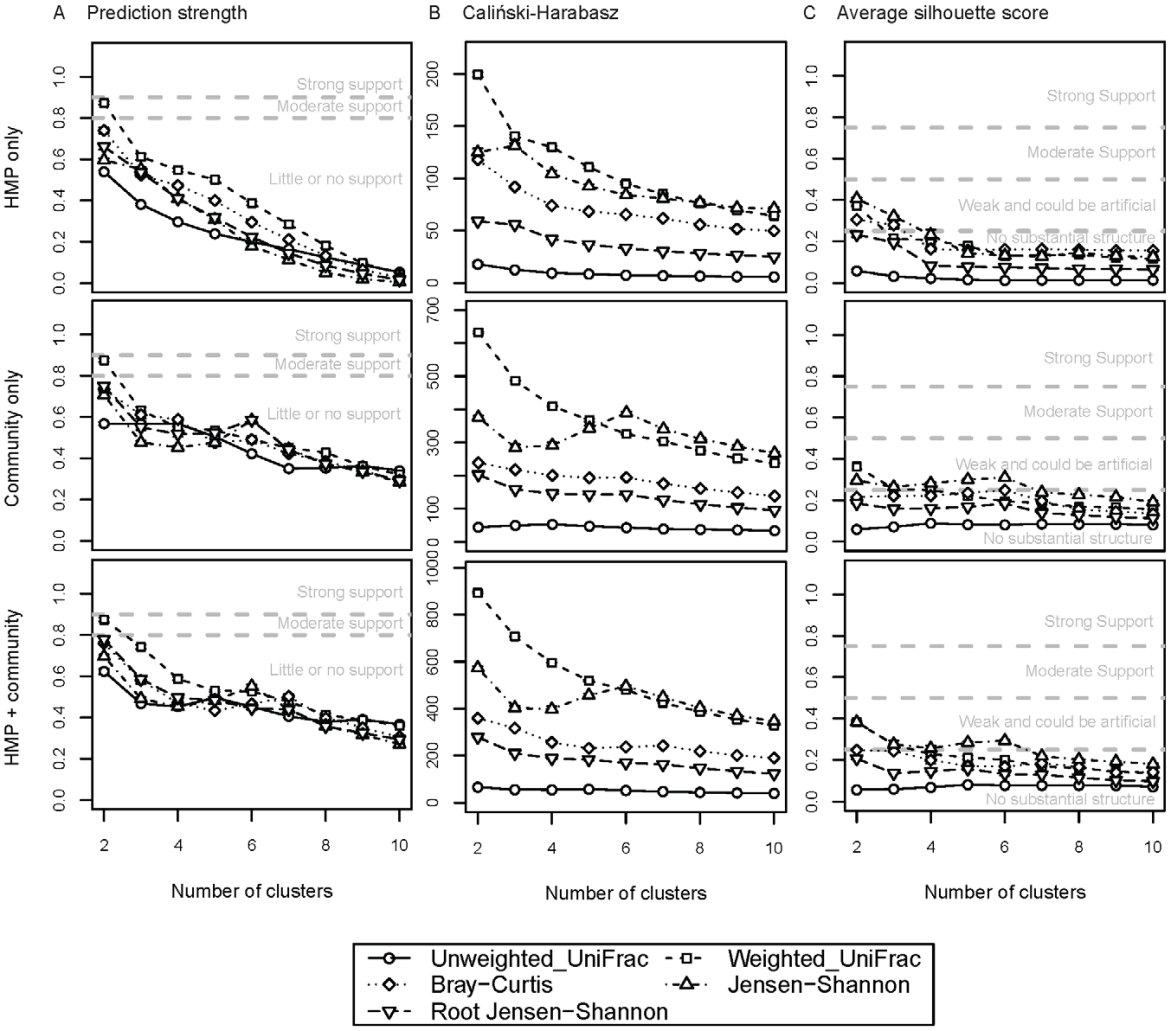
Clustering scores for enterotypes in fecal samples using 16S rRNA data. (A) Prediction strength scores, (B) Caliński-Harabasz and (C) average silhouette scores calculated using 5 distances metrics for HMP data only, adult community data, and combined HMP and adult community data. The thresholds for significance of clustering scores are indicated as dashed lines on the plots. Bars are standard errors.

We searched for fecal enterotypes in the HMP and community 16S rRNA gene sequence data using the relative abundances of OTUs across samples, and applying five different distance metrics: JSD, rJSD, BC, and weighted/unweighted UniFrac distances, and three cluster evaluation methods (PS, CH and SI; Fig. 3). Using PS, we observed at best moderate support for 2 fecal enterotypes in the HMP data using weighted UniFrac, but little or no support using other distance metrics (Fig. 3A). We obtained similar results using community data alone and when combined together (Fig. 3A). Weighted UniFrac scoring for enterotypes was weak with SI (Fig. 3). Figs. S3, S4, S5, S6, S7 show similar analyses for 3 different vaginal sites, and 9 oral and 3 skin sites. Moderate to strong clustering is evident in only 3 out of these 15 body sites. In the mid vagina there is strong support for 2 clusters (discussed below) using BC, JSD and rJSD (Fig. S3; weighted UniFrac provided moderate support; unweighted UniFrac provided no support). In the posterior fornix (Fig. S3) and the attached keratinized gingiva (Fig. S4), we observed moderate support for 2 clusters using 5 and 4 distance metrics, respectively (unweighted UniFrac resulted in little or no support in the gingiva).

These results indicate that the detection of enterotypes is sensitive to the distance metric used, a result also recently reported by Claesson *et al*. [25]. Note that this sensitivity is not dependent on body site. However, because enterotyping is driven by the relative abundances of specific genera within samples, unweighted UniFrac, which takes into account presence/absence of tree branches but not abundances of sequences mapping to those branches, may not be an ideal distance measure to use for enterotyping. We include it here because it is widely used in microbiome studies. In contrast, the weighted UniFrac, BC, JSD and rJSD distance metrics are based on OTU abundances and should in principle be more appropriate. The lack of concordance between results based on different abundance-based distance metrics raises the following questions: if enterotypes are to be considered robust, must they be observed using more than one distance metric? Or does the lack of concordance between results using different distance metrics indicate that (here at least) weighted UniFrac is the best choice for enterotyping? Because the interpretation of the findings is currently subjective, and in the absence of any community-wide best practices, we recommend using at least 2 or 3 distance metrics and clearly stating the criteria used for calling enterotypes within the context of any particular study. Particularly, if different metrics yield different results, authors should attempt to understand the discrepancies and justify their choice of distance metric.

### Effect of OTU Taxonomic Level

**Figure 4:**
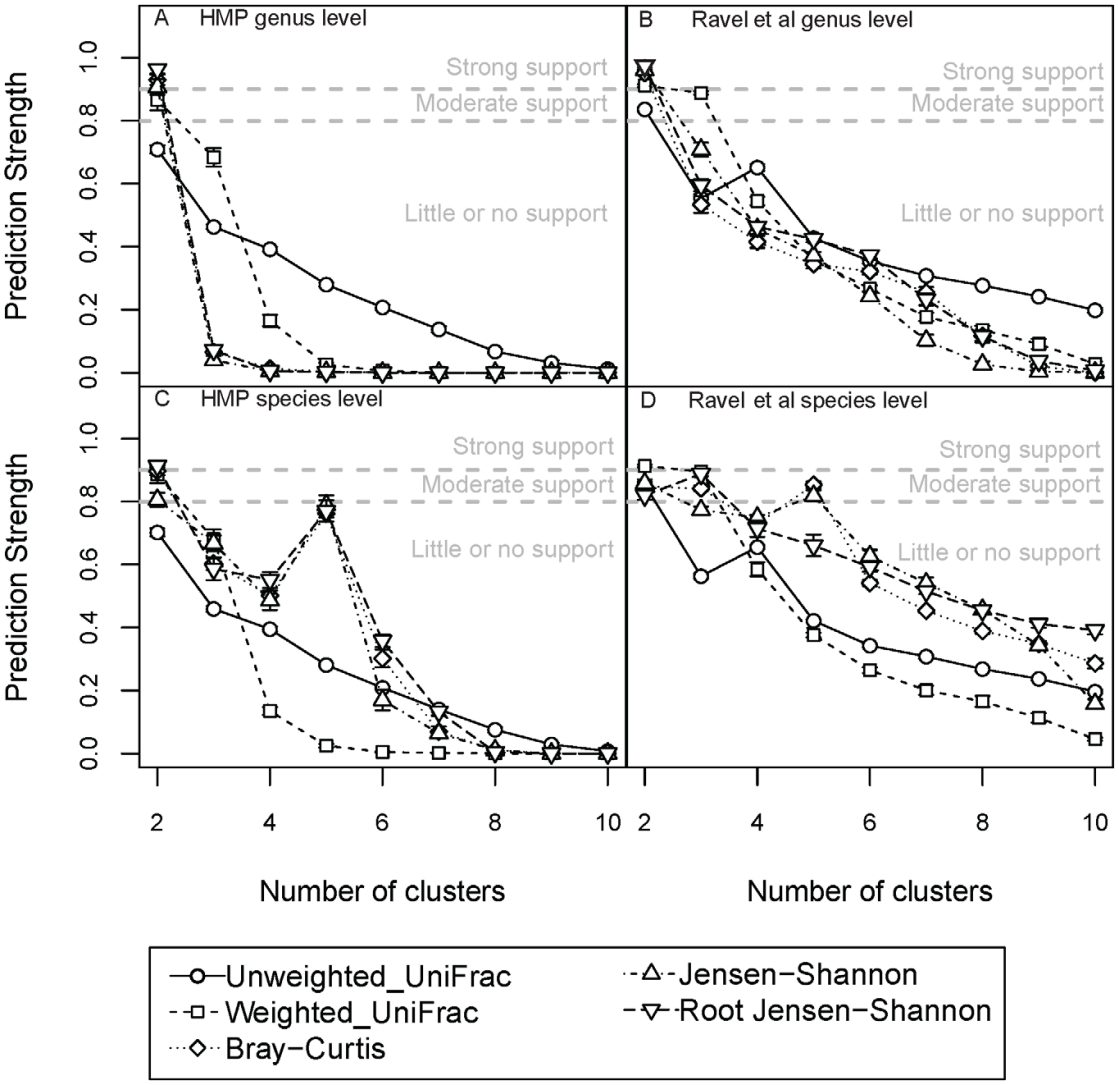
Enterotypes in mid vaginal samples in both the HMP and the Ravel Prediction strength scores calculated using 5 distances metrics for HMP mid vaginal samples at the genus level (A), Ravel *et al*. mid vaginal samples at the genus level (B). HMP mid vaginal samples at the species level (C) and Ravel *et al*. mid vaginal samples at the species level (D). The thresholds for significance of clustering scores are indicated as dashed lines on the plots. Bars are standard errors.

The effects of OTU taxonomic levels (for instance, clustering sequences at genus or species level) on the recovery of enterotypes are best illustrated with 16S rRNA gene sequence data from vaginal sites (Figs. 4 and S3). Ravel *et al.* [26] reported enterotypes in the vagina based on the abundances of bacterial species (as opposed to genera used in gut studies). We used the abundances of both species and genus-level OTUs from the Ravel *et al.* dataset to test for enterotypes. Our analysis shows strong support for two genus-level enterotypes using 4 of 5 distance metrics (*i.e.*, unweighted UniFrac had moderate support) for the Ravel *et al.* dataset when using the PS to test the strength of the clustering (Fig. 4). We also observed strong support for genus-level mid-vaginal enterotypes using 3 of 5 distance metrics (BC, JSD and rJSD) for the HMP dataset (Fig. 4). Additionally, using a species-level analysis, we obtained moderate support for five enterotypes using BC and JSD in the Ravel *et al.* data (we also scored strong support for 2 enterotypes with weighted UniFrac), and moderate to strong support for 2 clusters (*i.e.*, little or no support for five clusters) in the HMP data (Fig. 4).

We also tested for clustering of vaginal samples using SI and CH. When using SI (Fig. S8) at the genus level we found strong support for 2 clusters in the HMP and Ravel *et al.* datasets using 3 and 1 distance metrics respectively. But when using CH (Fig. S9) on the HMP data at the genus level, the highest scores were obtained for 2–3 and 9–10 clusters, and in the Ravel data the strongest support was for 2 clusters. At species level, we observed strong support with SI for 2 enterotypes in the HMP data using weighted UniFrac, and for 5 enterotypes using JSD (Fig. S8). No strong support was observed for the Ravel data for any number of clusters at the species level using SI. With CH, at the species level, the highest score was for 10 clusters in the HMP data, while for the Ravel data the highest score was for 2 clusters. The differences in number of enterotypes found at the genus and species levels underscore the sensitivity of enterotyping to the taxonomic depths used in constructing OTUs.

### Effect of 16S rRNA Variable Region

To test for the influence of the specific variable region of the 16S rRNA gene on the detection of fecal enterotypes, we compared fecal samples from the HMP for which sequence data for both the V1–V3 and V3–V5 regions were available. Data from the V3–V5 region yielded moderate support for two fecal enterotypes, but no enterotypes were detected using data from the V1–V3 region. When using SI, we observed moderate support for 2 clusters when using JSD on the V1–V3 data and weak support for the V3–V5 data. The highest scores using CH were three clusters using BC for V1–V3 data and two clusters using weighted UniFrac for V3–V5 data (Fig. S10). Different primers amplifying different regions of the 16S rRNA gene sequence are known to impact the diversity described for a microbial community. For example, primers for the V1–V3 region (*e.g.*, 27F-338R) are not efficient for amplifying 16S rRNA gene sequences from members of the *Bifidobacteria* genus, which can dominate the infant microbiota [22], [27]. Our analysis demonstrates that the specific region of the 16S rRNA gene that is amplified during PCR is another factor that can affect the outcome when searching for enterotypes.

### Effect of OTU-Picking Method

**Figure 5:**
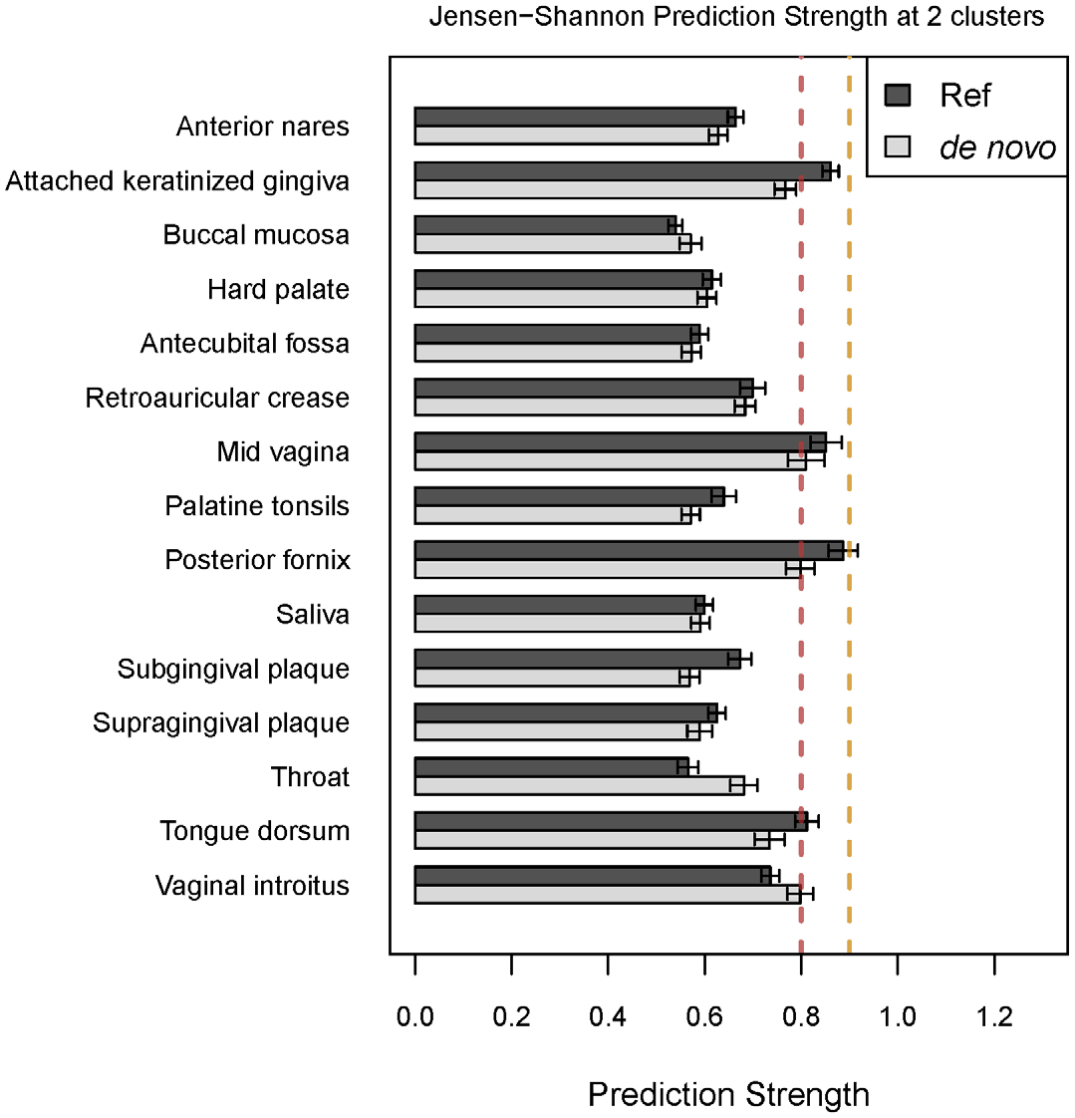
A comparison of prediction scores using different OTU picking methods. Prediction strength scores were calculated with JSD at 2 clusters using either OTUs generated using a reference-based approach or *de novo*.

We compared enterotype clustering using two methods for OTU picking: (1) *de novo* sequence clustering into OTUs, in which sequences are clustered based on similarity to one another, and (2) a reference based approach, in which sequences are clustered based on similarity to sequences in a reference database [27]. We found that for the HMP dataset, the two OTU picking approaches yielded consistent results for the majority of body sites (Fig. 5). However, for the attached keratinized gingiva, posterior fornix and tongue dorsum, the reference-based approach provided moderate support for enterotypes, whereas the *de novo* approach did not support clustering. One important difference between the two OTU-picking approaches is that the reference-based method can yield fewer OTUs, particularly at fine taxonomic resolution, because any sequence that fails to find a match in the database is discarded. In contrast, the *de novo* approach retains all sequences and has the potential to yield higher OTU counts. Fewer OTUs would have the effect of increasing the relative abundances of the dominant genera, and may therefore strengthen the gradient effect frequently observed (see below). Thus, the reference-based OTU picking approach may result in over-confidence in enterotype discovery.

### Effect of Sequence Rarefaction Depth

Rarefaction is the commonly used normalization practice of randomly subsampling the data so that an equal number of sequences are drawn for each sample. We rarefied the sequences from the HMP fecal samples at 2,000 sequences per sample and compared the results to those obtained after rarefying at 1,000 sequences per sample (Fig. S11). Rarefaction depth did not seem to strongly affect the results of the clustering.

### Effect of Data Type: WGS versus 16S rRNA Gene Sequences

**Figure 6:**
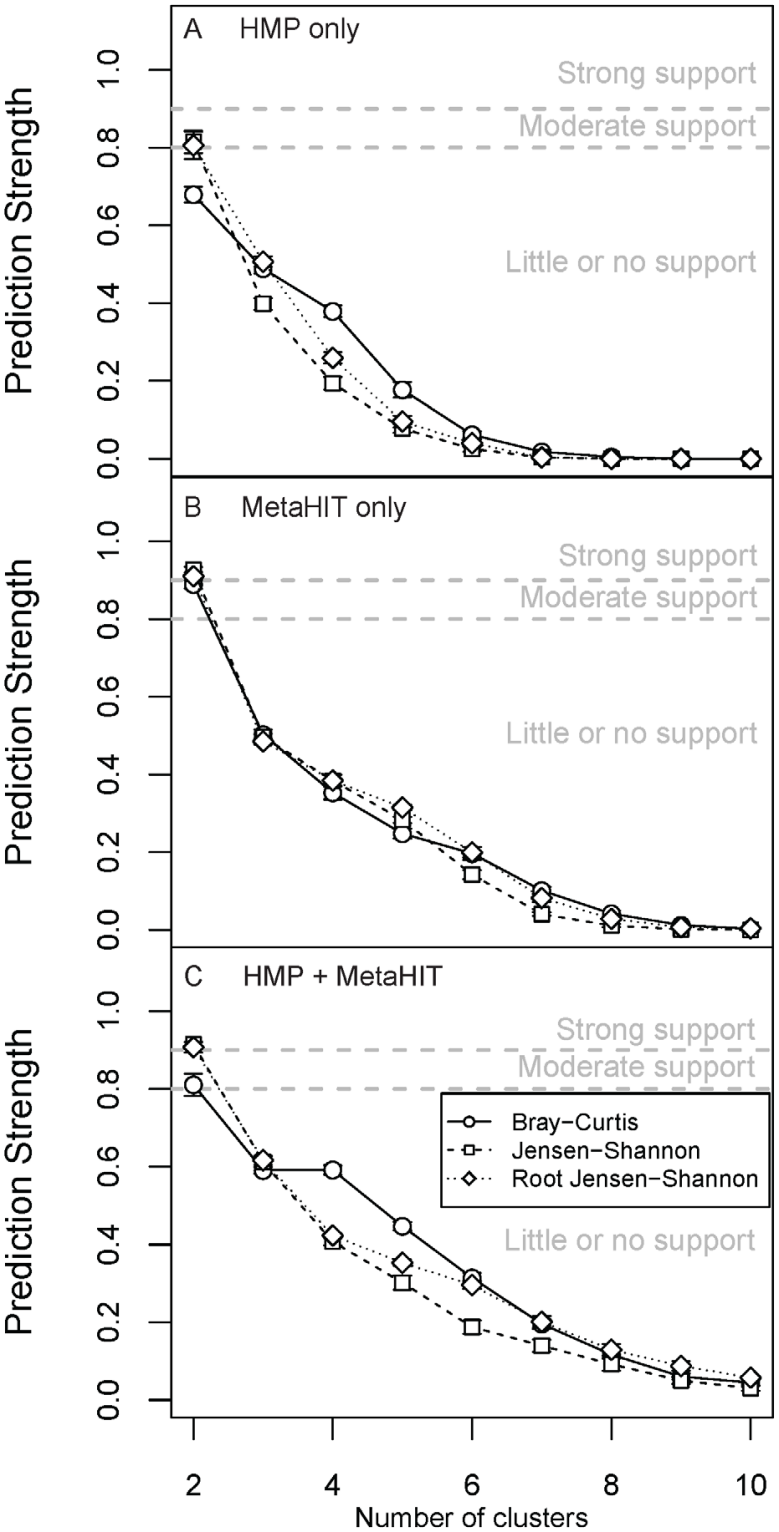
Prediction scores for enterotypes in fecal samples using WGS data. Prediction strength scores calculated using 3 distances metrics for (A) HMP, (B) MetaHIT and (C) HMP + MetaHIT data. The thresholds for significance of clustering scores are indicated as dashed lines on the plots. Bars are standard errors.

We also implemented our methodology in the smaller set of HMP samples for which WGS data were available, in addition to the MetaHIT WGS data [3]. While the HMP WGS data included fewer samples and body sites than the 16S rRNA gene sequence data (approximately 700 spanning the gut, nares, three oral habitats, and posterior fornix), they provided consistent species-level resolution. We found a strong gradient effect in the fecal samples (see discussion on gradients below) for almost all genera and species, and between species within the genus *Bacteroides* and members of the Firmicutes. We also found that the presence of *Prevotella* (specifically, *P. copri*) was clearly associated with the first principal coordinate in the PCoA using three distance measures. This feature in turn drove moderate support for two clusters in the HMP data (using JSD and rJSD) and strong support for 2 clusters in the MetaHIT data (Figs. 6 and S12), that appeared to separate roughly according to presence/absence of *Prevotella*.

*Prevotella* is similarly influential in driving variation along the first principal coordinate axis when using Jensen-Shannon divergence for the 16S-based samples, albeit not with weighted UniFrac (Figs. S13-S14). Although the importance of *Prevotella* in clustering analysis clearly depends on the choice of distance metric, the genus does exhibit enterotype-like behavior in that it follows a bimodal distribution: high relative abundance in a small fraction of samples, but low or zero relative abundance in many other samples. Note that the HMP 16S rRNA gene sequence surveys include a smaller fraction of samples containing high relative abundance of *Prevotella* compared to WGS data (12.3% and 10.9% of samples contained >10% *Prevotella* in the HMP V1–V3 and HMP V3–V5 data sets, respectively, compared to 13.9% and 24.2% in the HMP and MetaHIT shotgun metagenomics). Although a bias of certain primers against *Prevotella* in 16S surveys has been reported previously [28], this is not likely to have affected the HMP data. The difference in *Prevotella* abundance between MetaHIT and HMP samples remains to be explained.

In all other body sites, we again found general agreement between metagenomics clustering results and the 16S rRNA gene sequence-based clustering results regarding cluster quality (Figs. S15-S16), with the exception of the moderate support for two enterotypes in the buccal mucosa WGS data (Fig. S15), and lack of consistent support for enterotypes in the posterior fornix (Fig. S16). As we described above, we found moderate support for enterotypes in the posterior fornix in 16S data (Fig. S3). The discrepancy might be due to the fact that the WGS data included too few samples from the smaller “clusters” to permit detection by the prediction strength approach; SI was highest for two enterotypes at the genus level corresponding to *Lactobacillus* (either dominant or absent; JSD jackknifed SI: 0.89±0.012), and statistically tied as highest for five (four *Lactobacillus* species or *Lactobacillus* absent, JSD jackknifed SI: 0.79±0.005) and eight (JSD jackknifed SI: 0.79±0.008) enterotypes at the species level (Fig. S17).

### Gradients of OTU Abundances

#### *Bacteroidetes*/Firmicutes in gut samples

**Figure 7:**
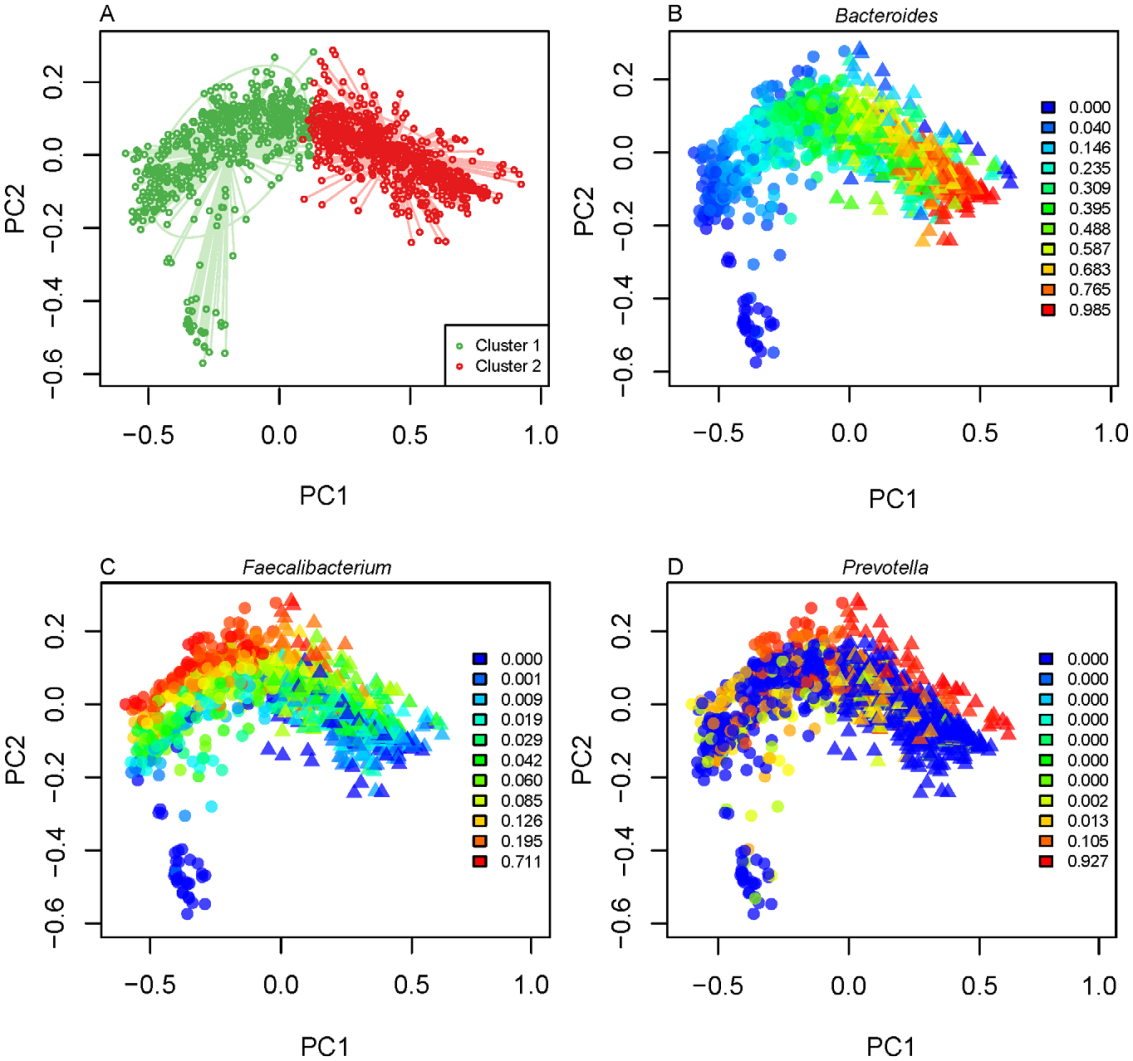
Gradients of OTU abundances are evident in the combined dataset of fecal samples. HMP and community fecal samples are shown in a PCoA of weighted UniFrac distances. Samples are colored according to (A) putative cluster membership and by their abundances (0–1, see legend inserts) of (B) *Bacteroides*, (C) *Faecalibacterium* and (D) *Prevotella*.

**Figure 8:**
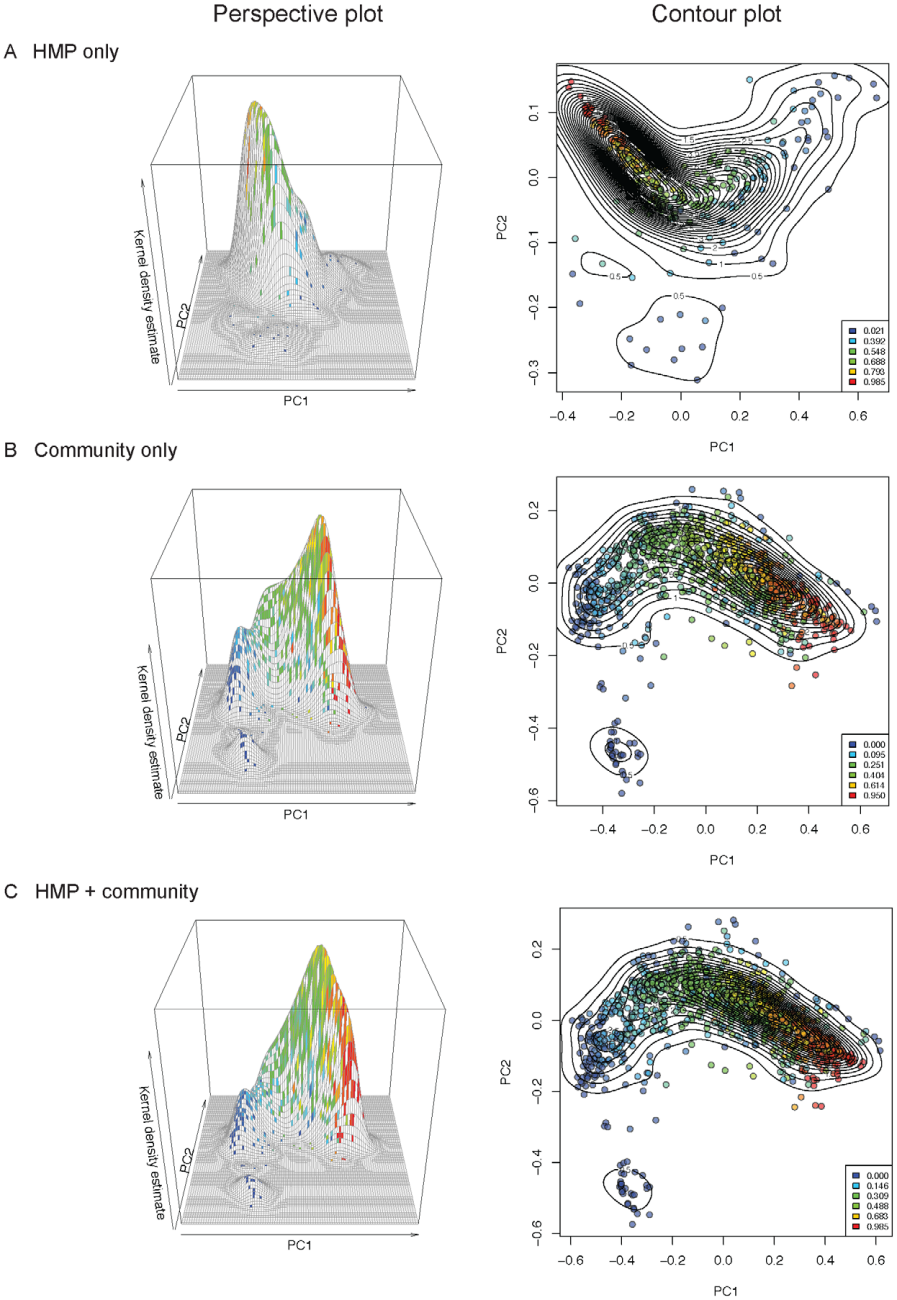
The fecal microbiota exhibit a smooth gradient of *Bacteroides* abundances across samples from the HMP and community studies. *Bacteroides* abundances are mapped onto the first two principal coordinates of the weighted UniFrac PCoA analysis for HMP data (A), community data (B), and combined HMP and community data (C). Left panels: 3D plots showing kernel density estimates mapped onto PC1 and PC2; Right panels: contours indicate sample densities, sample colors indicate *Bacteroides* relative abundances ranging from 0–1, where 1 = 100% *Bacteroides*; color levels are determined by quantiles to allow visual comparison of any distribution of relative abundances (*e.g.*, 0% of samples fall below the first threshold, 20% below the second threshold, 40% below the third, etc.)

**Figure 9:**
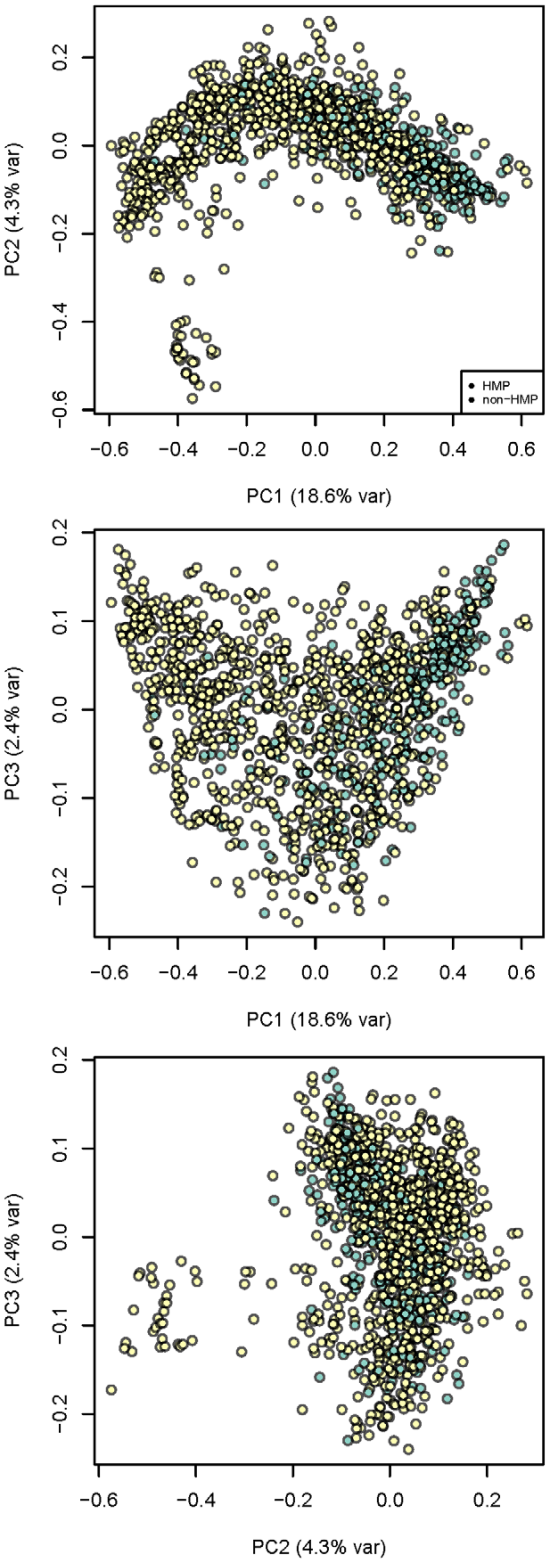
HMP fecal samples are slightly enriched in *Bacteroides* abundances compared to community samples. The projection from Fig. 8C, right panel, is colored to show if samples originate from the HMP (blue) or community (yellow), and all PC combinations are shown. See Fig. 8 for description of the axes.

Gut enterotypes would be apparent if the underlying data contained sharp divisions in the distribution of samples based on genus-level abundances (*i.e.*, clear regions with no samples between the modes in PCoA plots). We observed a smooth gradient distribution of HMP + community samples based on their *Bacteroides* abundances (Fig. 7), which in turn was the major determinant of inter-individual β-diversity patterns (Fig. 8). Samples at the extremes of this gradient were highly enriched in or depleted of *Bacteroides*, but such extremes can be interpretable as outliers on a continuum, rather than statistically significant groups. To further assess the possibility that this gradient was bi- or multi-modal, we mapped the density of samples along the first two dimensions of weighted UniFrac PCoA (Fig. 8). The kernel density estimates for samples mapped on principal coordinates 1 and 2 show two peaks emerging from the community data (but not from HMP data), which contributed to a second, low peak in the combined data (Fig. 8). The HMP samples tended to have higher *Bacteroides*-abundance when compared to the community samples (Fig. 9).

The vast majority of samples occupies the intermediate region between low- and high-*Bacteroides* individuals and forms a smooth distribution of gut community configurations (Fig. 8). Similar gradients of *Bacteroides* abundance across samples have been observed in other studies [3], [15]. The factors that drive the bimodal distribution of samples at ends of the gradient remain to be determined. Distinctive boundaries of gut enterotypes in small populations are likely to be blurred with the addition of samples containing intermediate levels of *Bacteroides*, although in some cases they may reflect host and environmental influences.

The extent to which a subject's microbiota varies along the gradient with time is not yet understood. Although many studies to date suggest relative stability of microbial communities over time within adult subjects [20], [29], [30], [31], the relative abundance of *Bacteroides* has been shown to vary over time within individuals, for instance, changes in *Bacteroides* abundances were reported over the course of a year-long weight loss study in obese individuals [30].

Recent work has also revealed an important role for nutrient status in determining the abundance of *Bacteroides*. Fasting (in mice), and feeding (in pythons) can alter the relative abundance of *Bacteroides* quite rapidly [32], [33]. In humans, excess energy intake above that needed for weight maintenance has been shown to reduce *Bacteroides* levels [34]. Furthermore, long-term dietary habits have been linked to enterotypes [15].

The low *Bacteroides*-abundance samples within the HMP + community dataset included a rare subset (total of 6 adult samples and 7 infant/child samples) with a high abundance (≥0.6) of *Prevotella* (Fig. S18), which was notable due to generally low abundance of this genus in most other samples (90.1% of adults had <10% *Prevotella*, 64.8% had none in V3–V5 region data rarefied at 1,000 sequences/sample). Samples with high *Prevotella* abundances have previously been observed in non-Western human populations [6]. This community pattern has also been suggested to associate with high-carbohydrate diets [15]. It is likely that the size of this *Prevotella* peak will increase with the addition of more data from diverse populations, especially given the fact that 7 of the 13 *Prevotella*-rich samples noted above came from non-western subjects. This *Prevotella*-dominant community, belonging to a distinct group of samples, was the most enterotype-like cluster we observed in the fecal samples.

#### Other body sites

We next looked for enterotypes within the oral samples, which had the greatest degree of beta-diversity and were also the richest (highest α-diversity) within the HMP population [2]. The 9 oral sites also contained gradients of OTU relative abundances (Figs. S19-S27). These tremendous ranges of phylogenetic diversity occurred in continua among individuals and did not provide strong support for discrete enterotypes (Fig. S4-S6), although in the attached keratinized gingiva we saw moderate support for two enterotypes using weighted UniFrac, JSD, rJSD and BC (Fig. S4).

We observed the same pattern was observed for the skin sites. Each of the three skin types contained typically one or two dominant OTUs that accounted for the majority of the genus-level abundances [2]. Our analysis did not detect any enterotypes in the skin sites (retroauricular creases, antecubital fossae and anterior nares; Fig. S7), as these communities contained gradients of genus abundances across samples (Figs. S28-S30).

### Prospectus

Our results underscore the importance of methodology in assessing whether populations can be categorized by enterotypes. Table 1 summarizes the factors that might have an effect on enterotyping: the two with the largest effect are the distance metric and the clustering score method. We recommend using at least one absolute scoring method (*i.e.*, PS or SI) combined with at least 2–3 distance metrics to verify the presence of enterotypes. When using the different scoring methods, authors should indicate and justify the choice of thresholds for indicating levels of support for enterotypes. Other factors that should be kept in mind are the data type (and if using 16S rRNA gene sequence data, the variable region sequenced) and the OTU picking method. At the present time, there is no community consensus on how to define an enterotype, and two researchers with the same data can easily come to opposite conclusions regarding the presence of enterotypes if they apply different criteria. Microbial ecologists and clinicians interested in the enterotype concept need to standardize enterotyping methods for the concept to gain utility.

**Table 1:** Summary of the factors that may affect enterotyping.

	Strength	Effect	No effect
Known effects	Strong	Distance metric	Rarefaction
	Strong	Cluster scoring method	
	Strong	Taxonomical level	
	Moderate	Variable region of the 16S rRNA	
	Weak	16S rRNA vs. WGS data	
	Weak	OTU picking method	
Potential effects to be tested		Sample processing method	
		Batch effects	
		PCR conditions	
		Study size	

The large size of the HMP dataset, augmented with the community and MetaHIT data, brought to light the extent of bacterial abundance gradients within body habitats. The presence of these gradients underscores that discrete enterotypes (*i.e.*, enterotypes with distinct boundaries) are lacking. Instead, for continuous OTU and genus gradients are the norm for most body sites, although a few body sites had multimodal distributions of samples with modes near the extremes of the gradients, and very few cases (*e.g.*, the vagina) had consistent discrete community types. The biological drivers of these patterns, and their robustness over time, may be manifestations of host-microbial interactions, especially if they correlate with host factors such as diet, lifestyle, or genetics.

## Supporting Information

Figure S1 Health state for the subjects whose samples are shown in Fig. 1. (A) All samples from healthy subjects, for all body sites. (B) Samples from individuals with health problems, or other factors that may influence the diversity of the microbiota (*i.e.*, smoking, use of antibiotics). (C) Combined data from panels A and B. For body site legend, see Fig. 1.
(TIFF)

Figure S2 The relative locations of the different studies containing gut samples in PCoA plots of weighted UniFrac and Jensen-Shannon distances (For study names see Table S1).
(TIFF)

Figure S3 Prediction strength scores for enterotypes in HMP (A) mid vagina, (B) posterior fornix, and (C) vaginal introitus samples. Prediction strength scores calculated using 5 distances metrics. The thresholds for significance of clustering scores are indicated as dashed lines on the plots. Bars are standard errors.
(ZIP)

Figure S4 Prediction strength scores for enterotypes in HMP (A) subgingival plaque, (B) supragingival plaque, and (C) attached keratinized gingiva samples. Prediction strength scores calculated using 5 distances metrics. The thresholds for significance of clustering scores are indicated as dashed lines on the plots.
(TIFF)

Figure S5 Prediction strength scores for enterotypes in HMP (A) buccal mucosa, (B) hard palate, and (C) tongue dorsum samples. Prediction strength scores calculated using 5 distances metrics. The thresholds for significance of clustering scores are indicated as dashed lines on the plots. Bars are standard errors.
(TIFF)

Figure S6 Prediction strength scores for enterotypes in HMP (A) saliva, (B) palatine tonsils, and (C) throat samples. Prediction strength scores calculated using 5 distances metrics. The thresholds for significance of clustering scores are indicated as dashed lines on the plots. Bars are standard errors.
(TIFF)

Figure S7 Prediction strength scores for enterotypes in HMP (A) retroauricular crease, (B) antecubital fossa, and (C) anterior nares samples. Prediction strength scores calculated using 5 distances metrics. The thresholds for significance of clustering scores are indicated as dashed lines on the plots. Bars are standard errors.
(TIFF)

Figure S8 Enterotypes in mid vaginal sites samples in both the HMP and the Ravel *et al*. [26] datasets. Average silhouette width scores calculated using 5 distances metrics for HMP mid vaginal samples at the genus level, Ravel *et al*. mid vaginal samples at the genus level, HMP mid vaginal samples at the species level, Ravel *et al*. mid vaginal samples at the species level. The thresholds for significance of clustering scores are indicated as dashed lines on the plots. Bars are standard errors.
(TIFF)

Figure S9 Enterotypes in mid vaginal sites samples in both the HMP and the Ravel *et al*. [26] datasets. Caliński-Harabasz scores calculated using 5 distances metrics for HMP mid vaginal samples at the genus level, Ravel *et al*. mid vaginal samples at the genus level, HMP mid vaginal samples at the species level, Ravel *et al*. mid vaginal samples at the species level. Bars are standard errors.
(TIFF)

Figure S10 Clustering scores for fecal enterotypes in data from (A) V1–V3 and (B) V3–V5 variable regions of the16S rRNA gene using PS, SI and CH. Clustering scores calculated using 5 distances metrics. The thresholds for significance of clustering scores are indicated as dashed lines on the plots. Bars are standard errors.
(TIFF)

Figure S11 Prediction scores for enterotypes in HMP fecal samples using 16S rRNA data and rarefying at (A) 1,000 and (B) 2,000 sequences per sample. Prediction strength scores calculated using 5 distances metrics. The thresholds for significance of clustering scores are indicated as dashed lines on the plots. Bars are standard errors.
(TIFF)

Figure S12 Clustering scores for enterotypes in MetaHIT fecal samples using WGS data. (A) Prediction strength scores, (B) average silhouette scores and (C) Caliński-Harabasz calculated using 3 distances metrics. The thresholds for significance of clustering scores are indicated as dashed lines on the plots. Bars are standard errors.
(TIFF)

Figure S13 Gradients of the 24 taxa most highly correlated with the first 2 PCs in HMP fecal samples using Jensen-Shannon divergence for the V3–V5 16S-based data.
(TIFF)

Figure S14 Gradients of the 24 taxa most highly correlated with the first 2 PCs in HMP fecal samples using weighted UniFrac for the V3–V5 16S-based data.
(TIFF)

Figure S15 Prediction scores for enterotypes in HMP samples using WGS data. Prediction strength scores calculated using 3 distances metrics for buccal mucosa (A), supragingival (B), and tongue dorsum (C) samples. The thresholds for significance of clustering scores are indicated as dashed lines on the plots. Bars are standard errors.
(TIFF)

Figure S16 Prediction scores for enterotypes in HMP samples using WGS data. Prediction strength scores calculated using 3 distances metrics for anterior nares (A), and posterior fornix (B) samples. The thresholds for significance of clustering scores are indicated as dashed lines on the plots. Bars are standard errors.
(TIFF)

Figure S17 Silhouette index for enterotypes in HMP vaginal samples using WGS data at different taxonomic levels. Silhouette index scores calculated using 3 distances metrics for genus (A), and species (B) levels. The thresholds for significance of clustering scores are indicated as dashed lines on the plots. Bars are standard errors.
(TIFF)

Figure S18 Taxon distribution for the 10 most common genera in the small number (13) of high *Prevotella* samples (≥0.6 relative abundance).
(TIFF)

Figure S19 Gradients of *Prevotella*, *Fusobacterium* and *Treponema* abundances in subgingival plaque samples. HMP samples are shown in a principal coordinates analysis of unweighted UniFrac distances. Samples are colored according to (A) putative cluster membership and by their abundances (0–1, see legend inserts) of (B) *Prevotella*, (C) *Fusobacterium* and (D) *Treponema*.
(TIFF)

Figure S20 Gradients of *Selenomonas*, *Streptococcus* and *Leptotrichia* abundances in supragingival plaque samples. HMP samples are shown in a principal coordinates analysis of unweighted UniFrac distances. Samples are colored according to (A) putative cluster membership and by their abundances (0–1, see legend inserts) of (B) *Selenomonas*, (C) *Streptococcus* and (D) *Leptotrichia*.
(TIFF)

Figure S21 Gradients of *Prevotella*, *Streptococcus* and *Porphyromonas* abundances in attached keratinized gingiva samples. HMP samples are shown in a principal coordinates analysis of unweighted UniFrac distances. Samples are colored according to (A) putative cluster membership and by their abundances (0–1, see legend inserts) of (B) *Prevotella*, (C) *Streptococcus* and (D) *Porphyromonas*.
(TIFF)

Figure S22 Gradients of *Haemophilus*, *Streptococcus* and *Veillonella* abundances in buccal mucosa samples. HMP samples are shown in a principal coordinates analysis of unweighted UniFrac distances. Samples are colored according to (A) putative cluster membership and by their abundances (0–1, see legend inserts) of (B) *Haemophilus*, (C) *Streptococcus* and (D) *Veillonella*.
(TIFF)

Figure S23 Gradients of *Streptococcus*, *Prevotella* and *Porphyromonas* abundances in hard palate samples. HMP samples are shown in a principal coordinates analysis of unweighted UniFrac distances. Samples are colored according to (A) putative cluster membership and by their abundances (0–1, see legend inserts) of (B) *Streptococcus*, (C) *Prevotella* and (D) *Porphyromonas*.
(TIFF)

Figure S24 Gradients of *Streptococcus*, *Porphyromonas* and *Atopobium* abundances in tongue dorsum samples. HMP samples are shown in a principal coordinates analysis of unweighted UniFrac distances. Samples are colored according to (A) putative cluster membership and by their abundances (0–1, see legend inserts) of (B) *Streptococcus*, (C) *Porphyromonas* and (D) *Atopobium*.
(TIFF)

Figure S25 Gradients of *Prevotella*, Unclassified Veillonellaceae and *Streptococcus* abundances in saliva samples. HMP samples are shown in a principal coordinates analysis of unweighted UniFrac distances. Samples are colored according to (A) putative cluster membership and by their abundances (0–1, see legend inserts) of (B) *Prevotella*, (C) Unclassified Veillonellaceae and (D) *Streptococcus*.
(TIFF)

Figure S26 Gradients of *Prevotella*, *Streptococcus* and *Porphyromonas* abundances in palatine tonsils samples. HMP samples are shown in a principal coordinates analysis of unweighted UniFrac distances. Samples are colored according to their (A) putative cluster membership and by abundances (0–1, see legend inserts) of (B) *Prevotella*, (C) *Streptococcus* and (D) *Porphyromonas*.
(TIFF)

Figure S27 Gradients of *Prevotella*, *Neisseria* and Unclassified Veillonellaceae abundances in throat samples. HMP samples are shown in a principal coordinates analysis of unweighted UniFrac distances. Samples are colored according to (A) putative cluster membership and by their abundances (0–1, see legend inserts) of (B) *Prevotella*, (C) *Neisseria* and (D) Unclassified Veillonellaceae.
(TIFF)

Figure S28 Gradients of *Propionibacterium*, *Staphylococcus* and *Corynebacterium* abundances in retroauricular crease samples. HMP samples are shown in a principal coordinates analysis of unweighted UniFrac distances. Samples are colored according to (A) putative cluster membership and by their abundances (0–1, see legend inserts) of (B) *Propionibacterium*, (C) *Staphylococcus* and (D) *Corynebacterium*.
(TIFF)

Figure S29 Gradients of *Propionibacterium*, *Staphylococcus* and *Parabacteroides* abundances in antecubital fossa samples. HMP samples are shown in a principal coordinates analysis of unweighted UniFrac distances. Samples are colored according to (A) putative cluster membership and by their abundances (0–1, see legend inserts) of (B) *Propionibacterium*, (C) *Staphylococcus* and (D) *Parabacteroides*.
(TIFF)

Figure S30 Gradients of *Corynebacterium*, *Staphylococcus* and *Propionibacterium* abundances in anterior nares samples. HMP samples are shown in a principal coordinates analysis of unweighted UniFrac distances. Samples are colored according to (A) putative cluster membership and by their abundances (0–1, see legend inserts) of (B) *Corynebacterium*, (C) *Staphylococcus* and (D) *Propionibacterium*.
(TIFF)

Table S1 List of studies used in the data analysis.
(DOCX)
