# Supplementary figures and images for: A Guide to Enterotypes across the Human Body: Meta-Analysis of Microbial Community Structures in Human Microbiome Datasets
Source: PLoS Comput Biol. 2013 Jan 10;9(1):e1002863. doi: 10.1371/journal.pcbi.1002863 (PMC3542080; doi:10.1371/journal.pcbi.1002863)

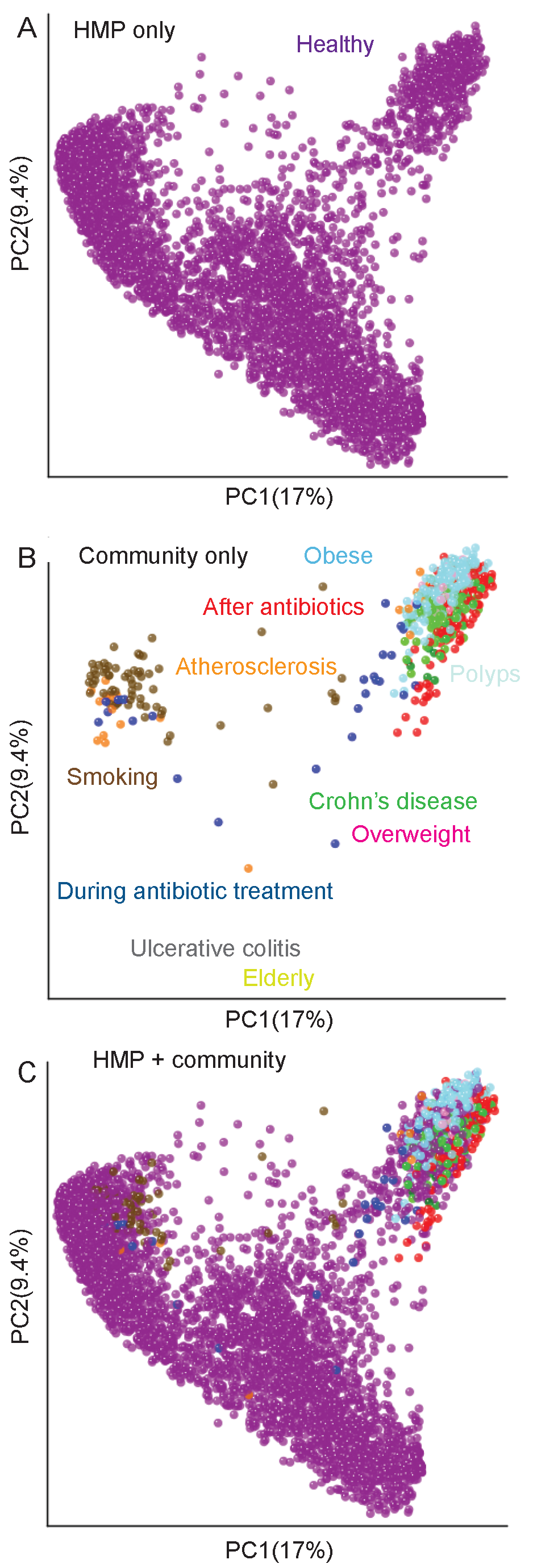

Supplement: Figure S1 — Health state for the subjects whose samples are shown in Fig. 1. (A) All samples from healthy subjects, for all body sites. (B) Samples from individuals with health problems, or other factors that may influence the diversity of the microbiota (i.e., smoking, use of antibiotics). (C) Combined data from panels A and B. For body site legend, see Fig. 1. (TIFF) [file pcbi.1002863.s001.tiff]

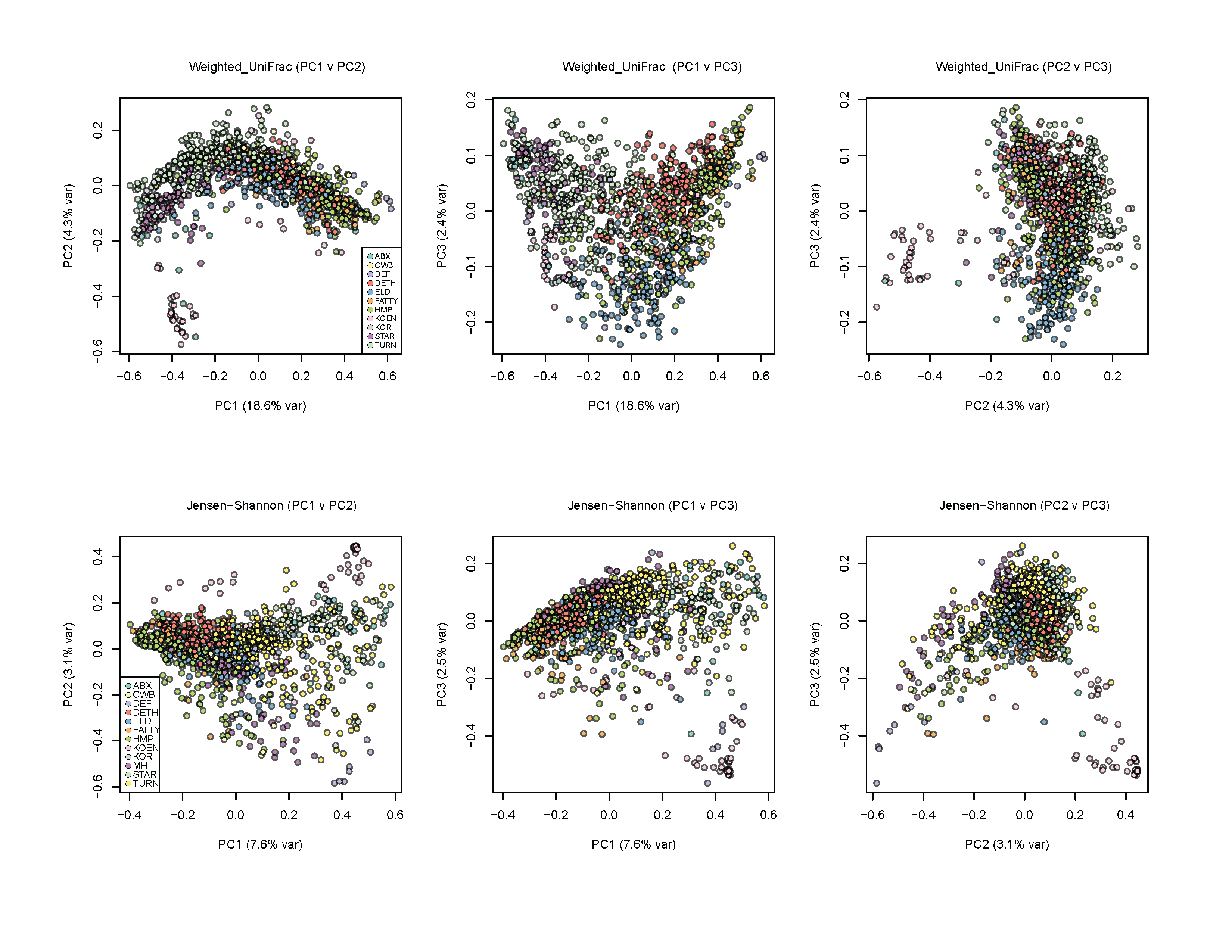

Supplement: Figure S2 — The relative locations of the different studies containing gut samples in PCoA plots of weighted UniFrac and Jensen-Shannon distances (For study names see Table S1). (TIFF) [file pcbi.1002863.s002.tiff]

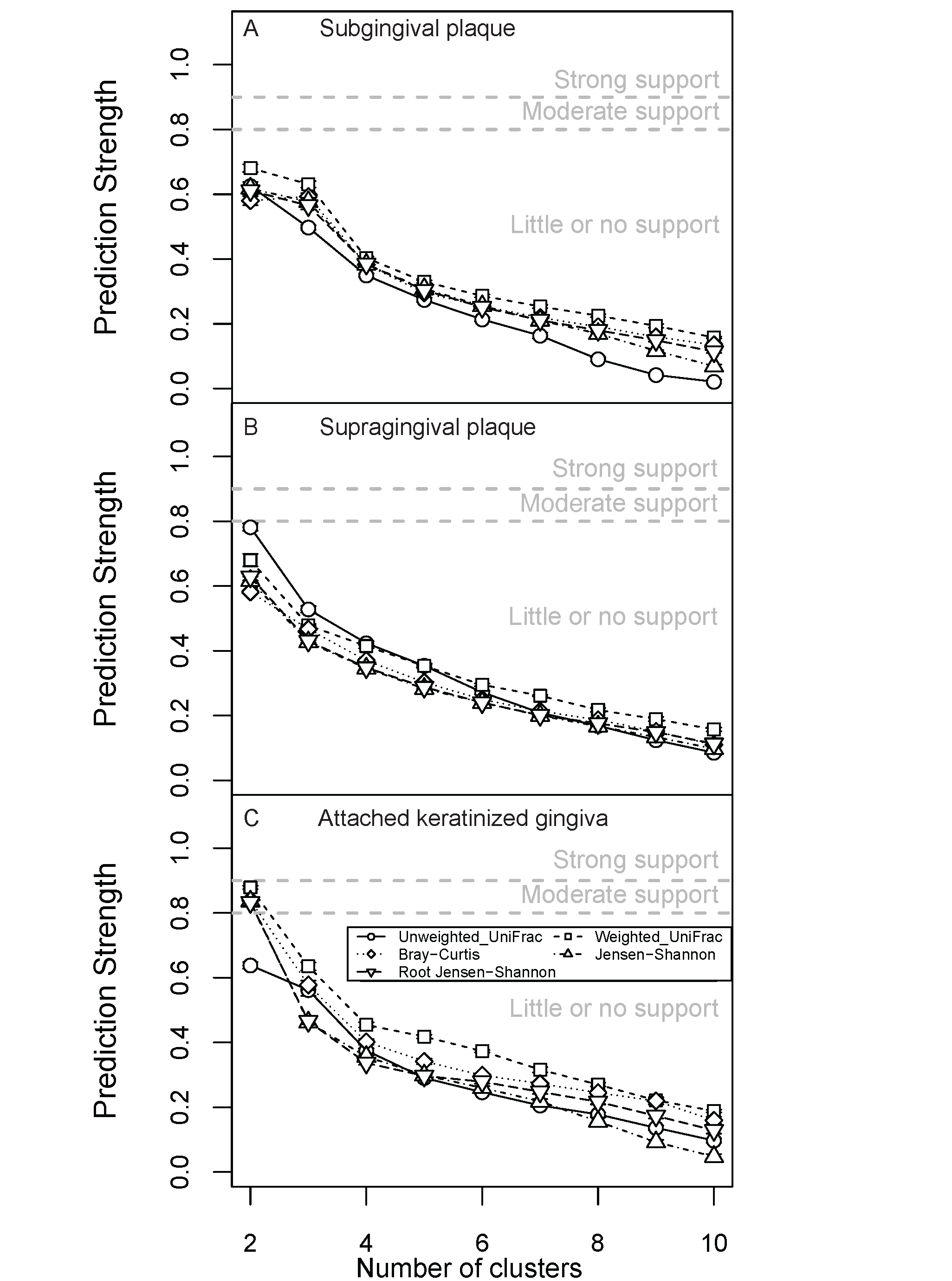

Supplement: Figure S4 — Prediction strength scores for enterotypes in HMP (A) subgingival plaque, (B) supragingival plaque, and (C) attached keratinized gingiva samples. Prediction strength scores calculated using 5 distances metrics. The thresholds for significance of clustering scores are indicated as dashed lines on the plots. (TIFF) [file pcbi.1002863.s004.tiff]

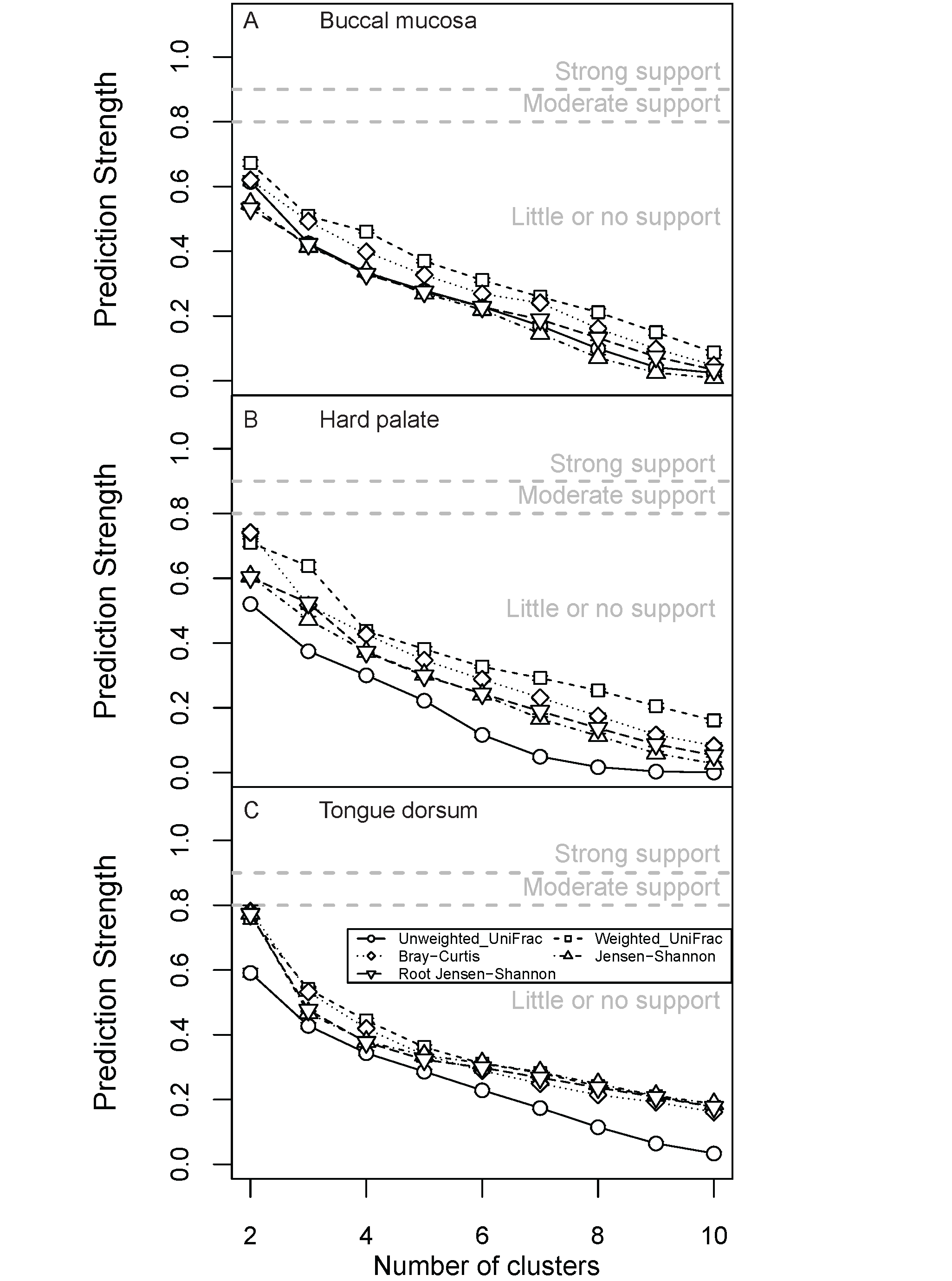

Supplement: Figure S5 — Prediction strength scores for enterotypes in HMP (A) buccal mucosa, (B) hard palate, and (C) tongue dorsum samples. Prediction strength scores calculated using 5 distances metrics. The thresholds for significance of clustering scores are indicated as dashed lines on the plots. Bars are standard errors. (TIFF) [file pcbi.1002863.s005.tiff]

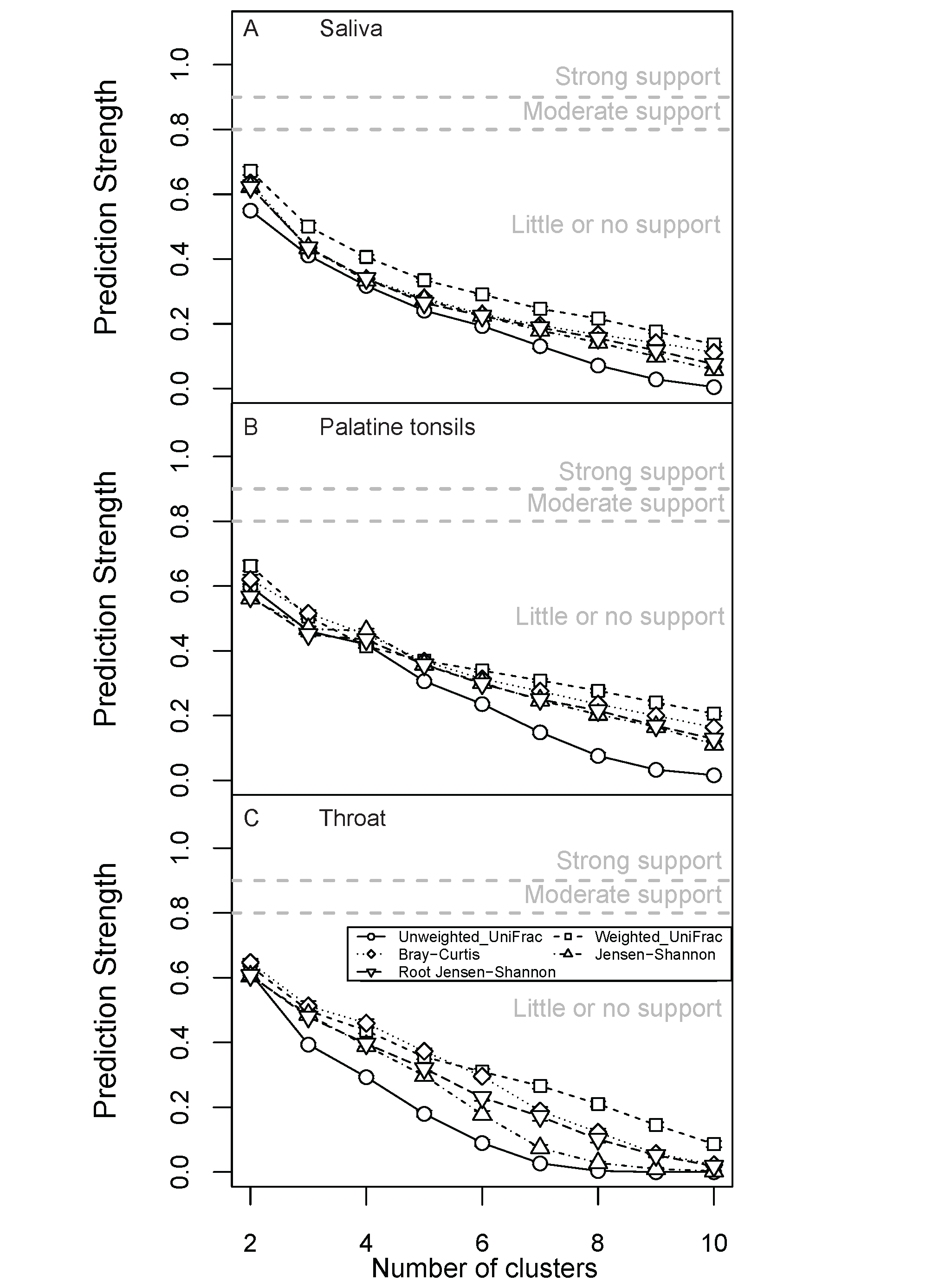

Supplement: Figure S6 — Prediction strength scores for enterotypes in HMP (A) saliva, (B) palatine tonsils, and (C) throat samples. Prediction strength scores calculated using 5 distances metrics. The thresholds for significance of clustering scores are indicated as dashed lines on the plots. Bars are standard errors. (TIFF) [file pcbi.1002863.s006.tiff]

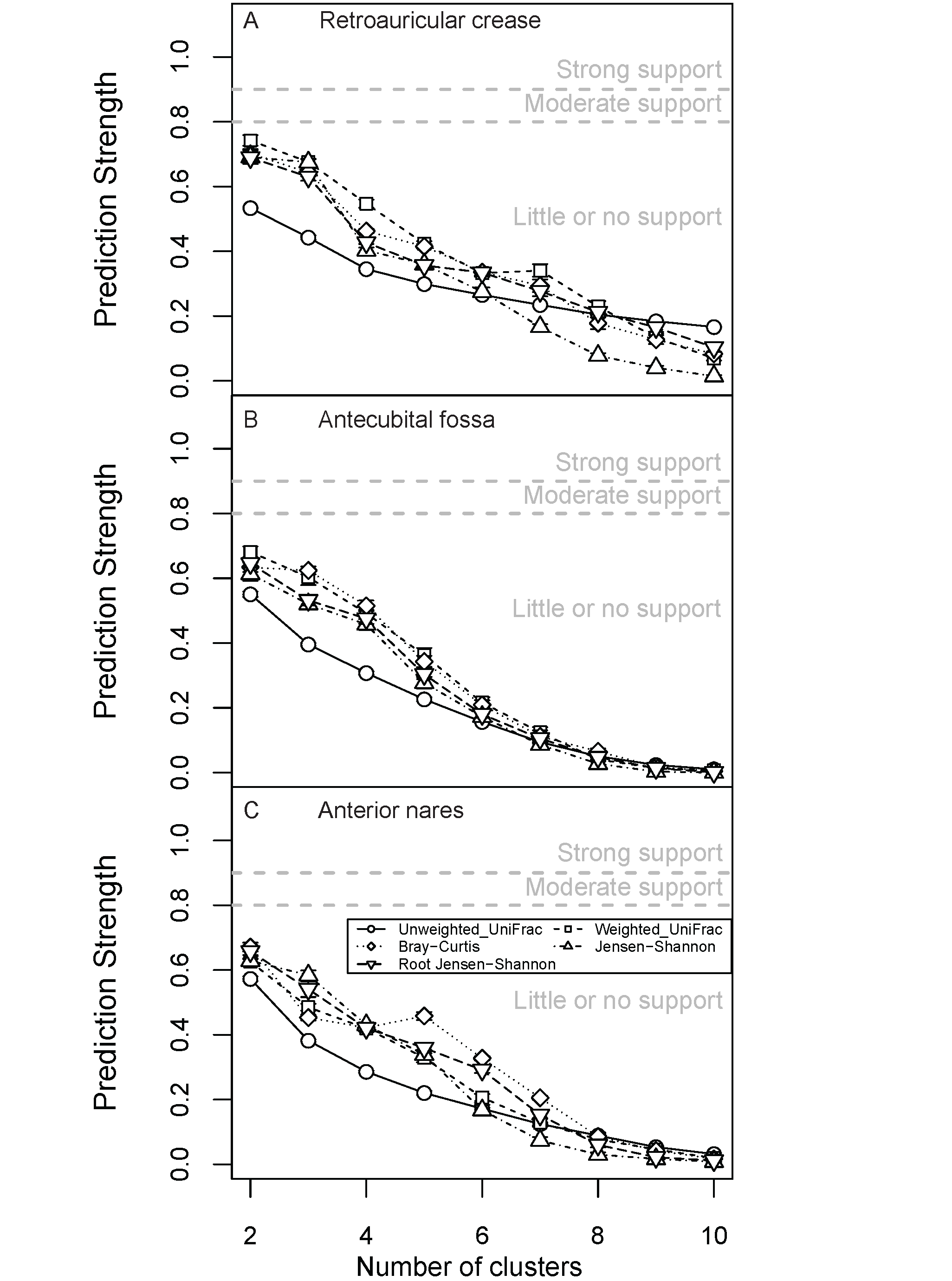

Supplement: Figure S7 — Prediction strength scores for enterotypes in HMP (A) retroauricular crease, (B) antecubital fossa, and (C) anterior nares samples. Prediction strength scores calculated using 5 distances metrics. The thresholds for significance of clustering scores are indicated as dashed lines on the plots. Bars are standard errors. (TIFF) [file pcbi.1002863.s007.tiff]

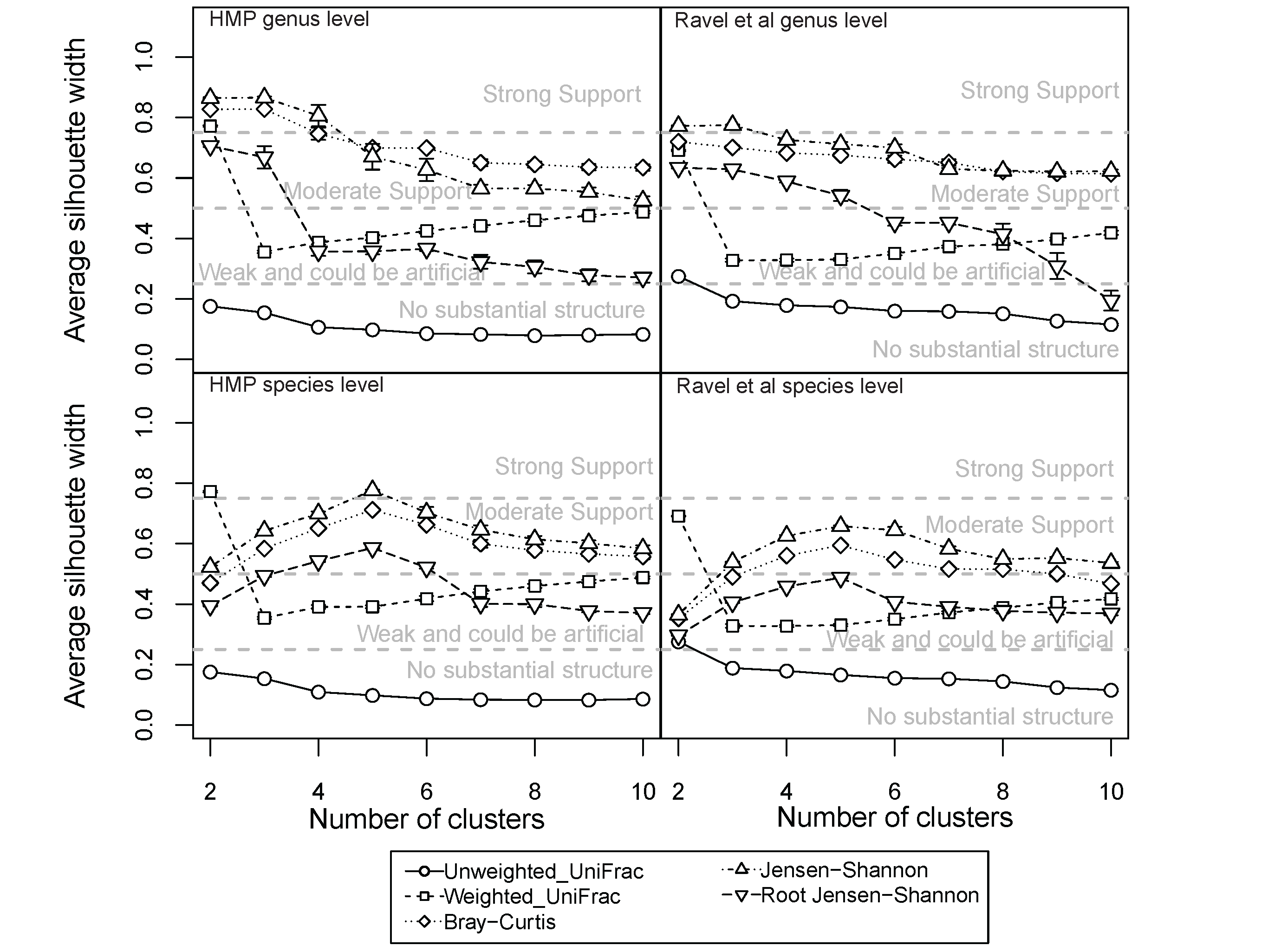

Supplement: Figure S8 — Enterotypes in mid vaginal sites samples in both the HMP and the Ravel et al. [26] datasets. Average silhouette width scores calculated using 5 distances metrics for HMP mid vaginal samples at the genus level, Ravel et al. mid vaginal samples at the genus level, HMP mid vaginal samples at the species level, Ravel et al. mid vaginal samples at the species level. The thresholds for significance of clustering scores are indicated as dashed lines on the plots. Bars are standard errors. (TIFF) [file pcbi.1002863.s008.tiff]

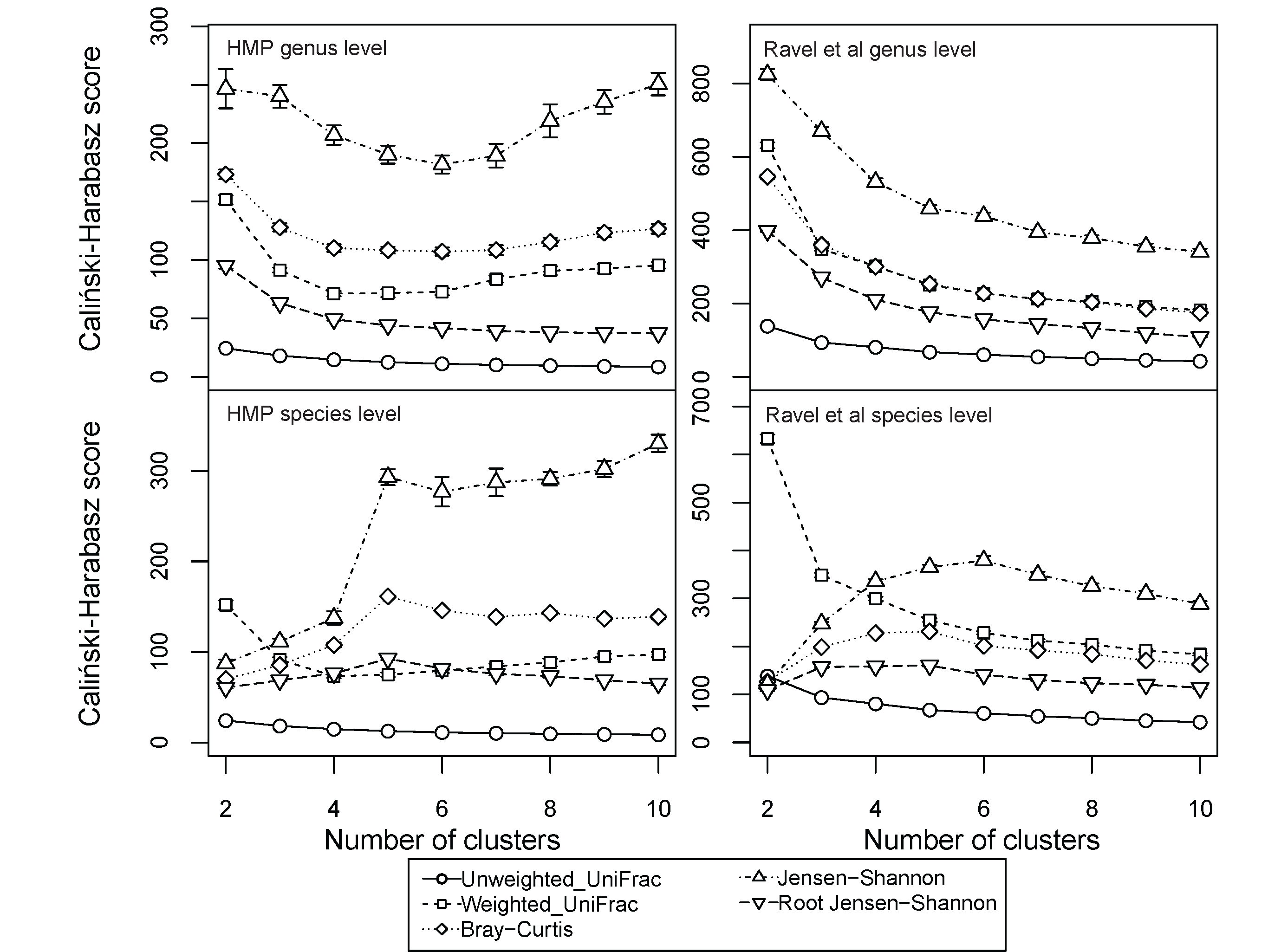

Supplement: Figure S9 — Enterotypes in mid vaginal sites samples in both the HMP and the Ravel et al. [26] datasets. Caliński-Harabasz scores calculated using 5 distances metrics for HMP mid vaginal samples at the genus level, Ravel et al. mid vaginal samples at the genus level, HMP mid vaginal samples at the species level, Ravel et al. mid vaginal samples at the species level. Bars are standard errors. (TIFF) [file pcbi.1002863.s009.tiff]

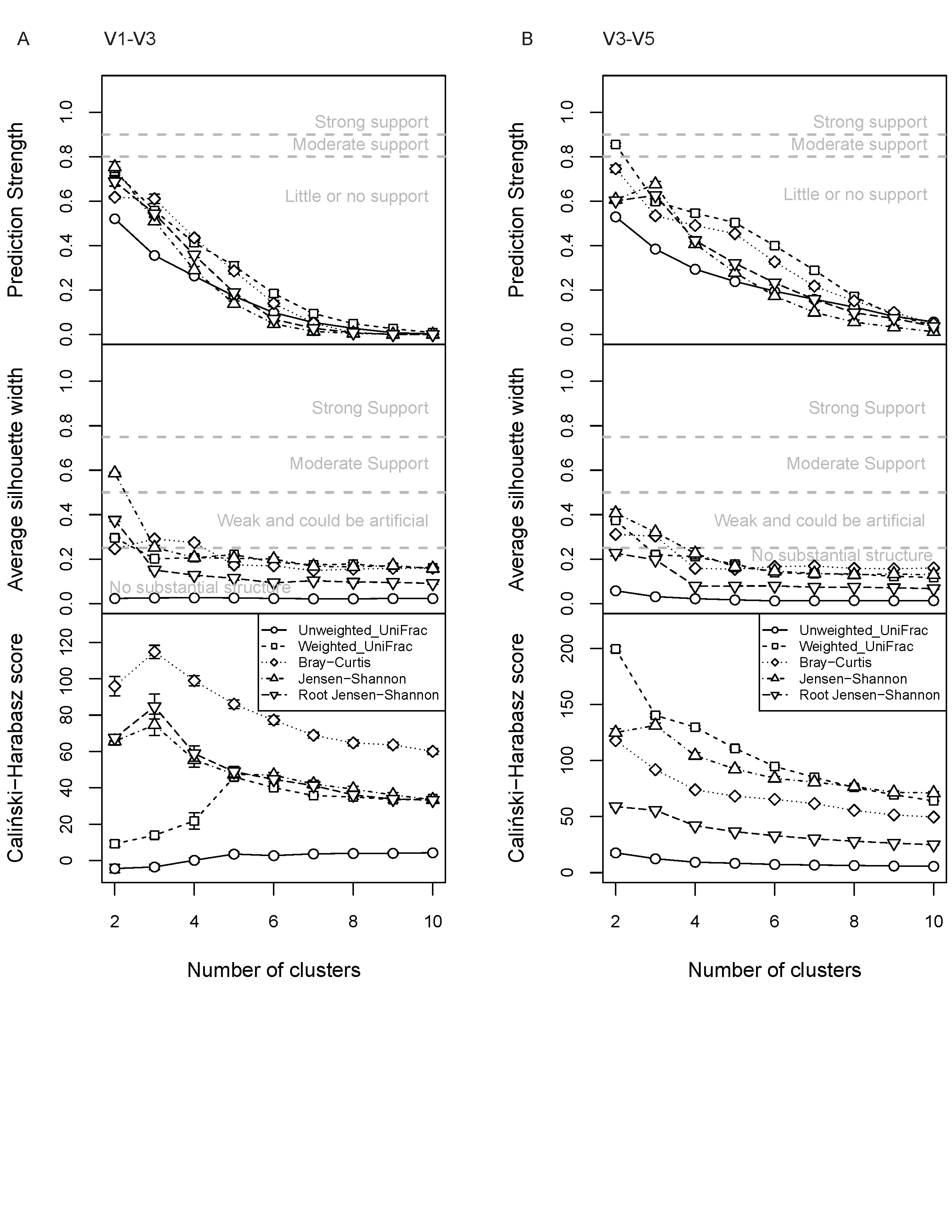

Supplement: Figure S10 — Clustering scores for fecal enterotypes in data from (A) V1–V3 and (B) V3–V5 variable regions of the16S rRNA gene using PS, SI and CH. Clustering scores calculated using 5 distances metrics. The thresholds for significance of clustering scores are indicated as dashed lines on the plots. Bars are standard errors. (TIFF) [file pcbi.1002863.s010.tiff]

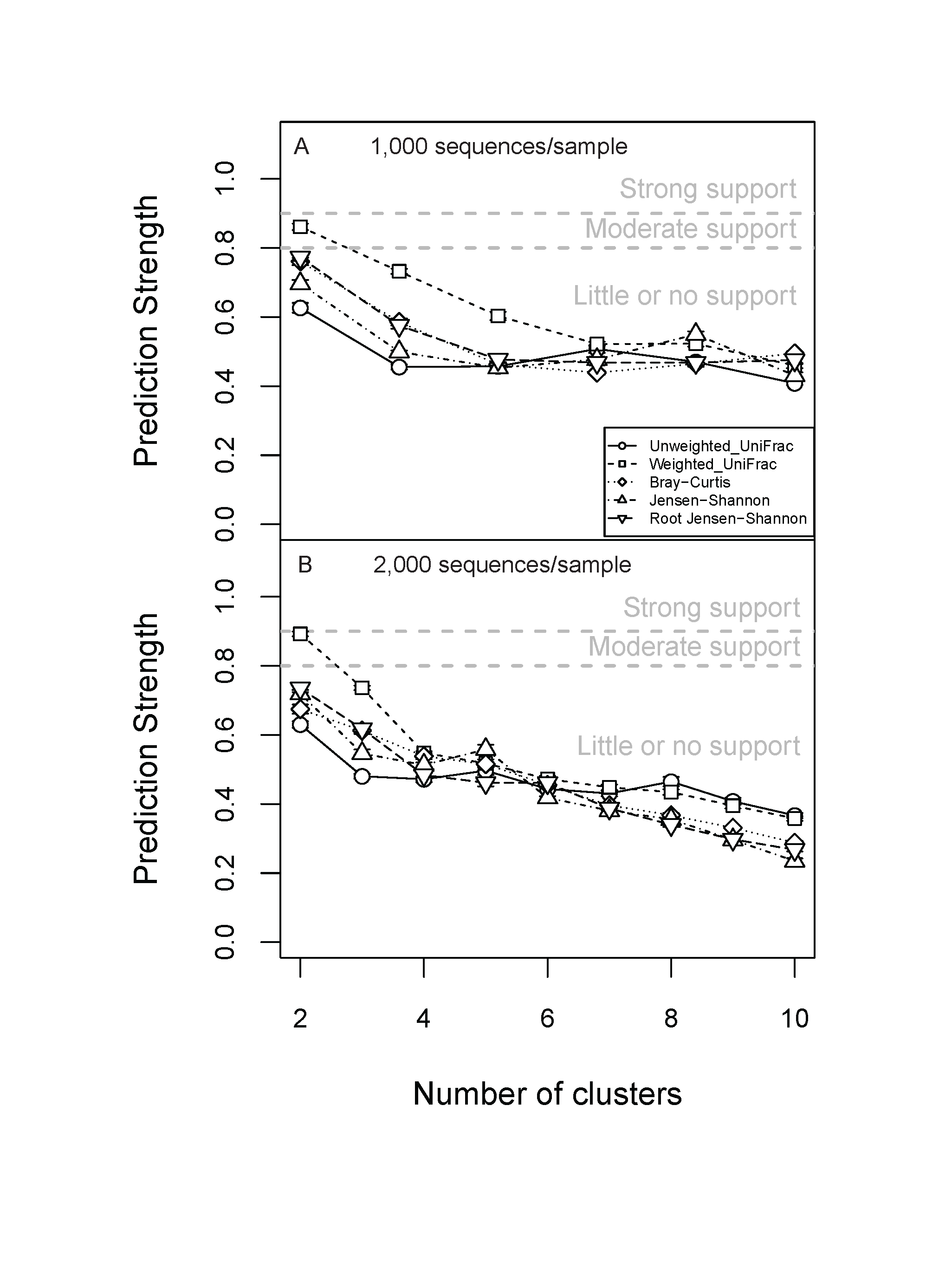

Supplement: Figure S11 — Prediction scores for enterotypes in HMP fecal samples using 16S rRNA data and rarefying at (A) 1,000 and (B) 2,000 sequences per sample. Prediction strength scores calculated using 5 distances metrics. The thresholds for significance of clustering scores are indicated as dashed lines on the plots. Bars are standard errors. (TIFF) [file pcbi.1002863.s011.tiff]

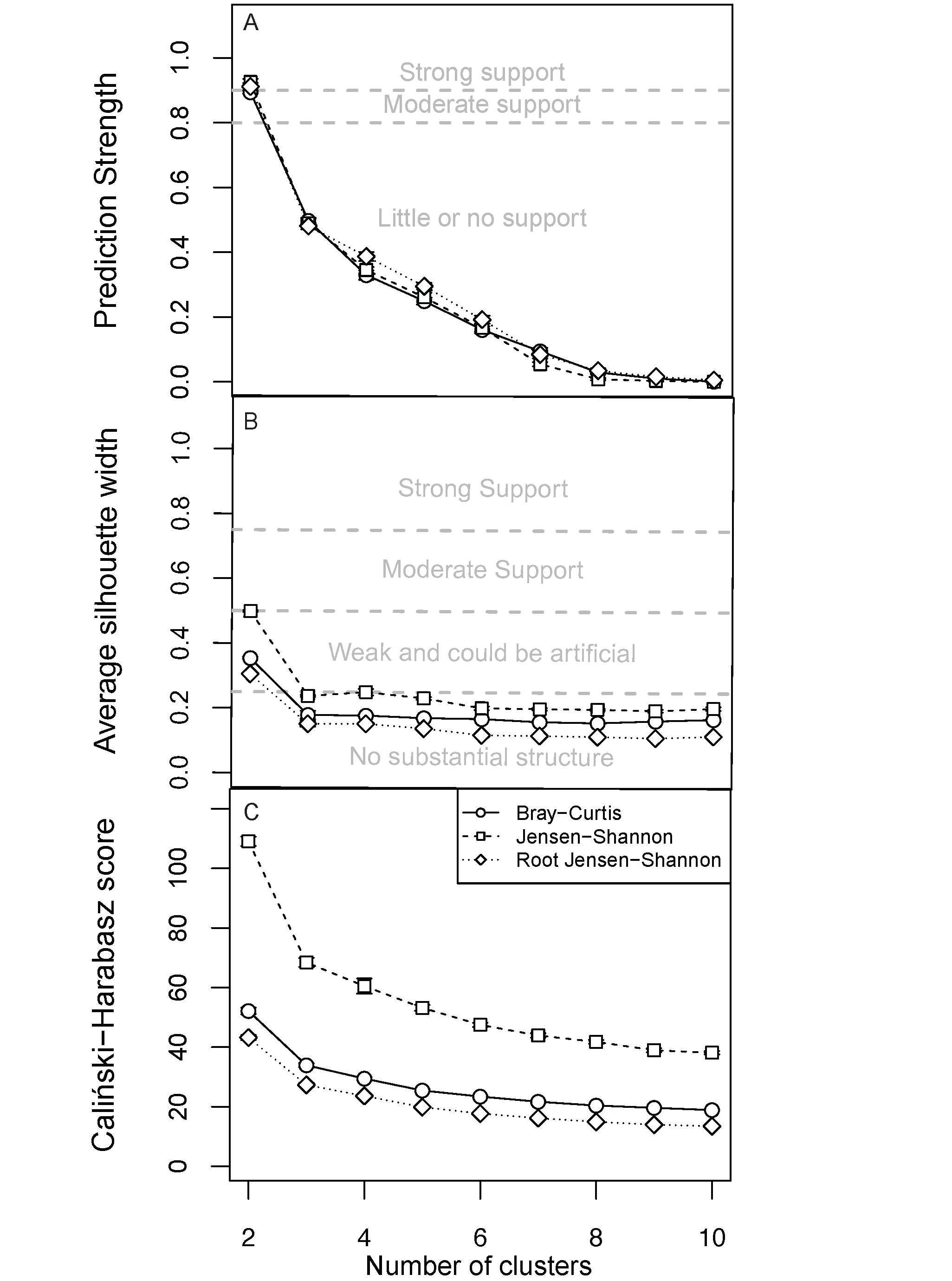

Supplement: Figure S12 — Clustering scores for enterotypes in MetaHIT fecal samples using WGS data. (A) Prediction strength scores, (B) average silhouette scores and (C) Caliński-Harabasz calculated using 3 distances metrics. The thresholds for significance of clustering scores are indicated as dashed lines on the plots. Bars are standard errors. (TIFF) [file pcbi.1002863.s012.tiff]

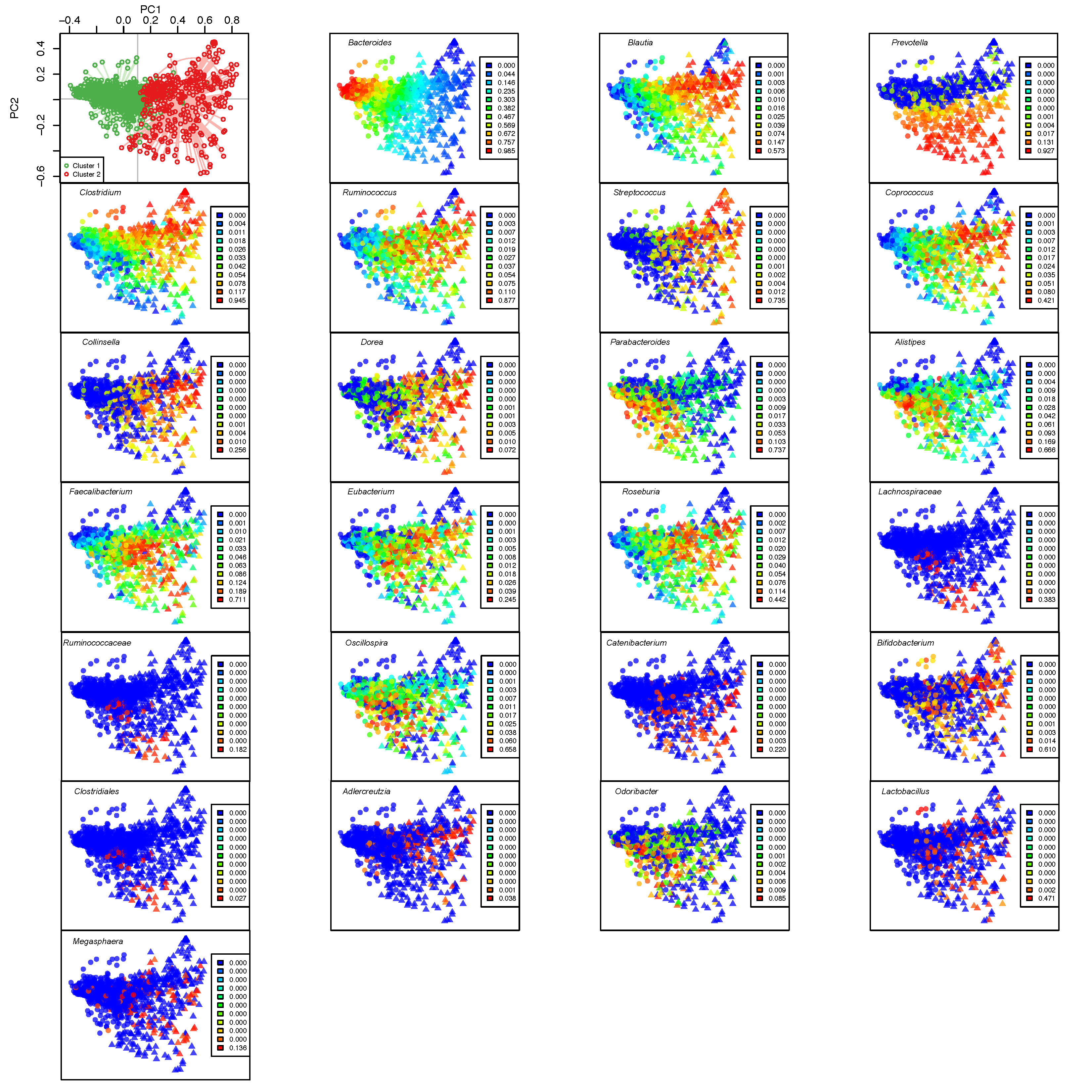

Supplement: Figure S13 — Gradients of the 24 taxa most highly correlated with the first 2 PCs in HMP fecal samples using Jensen-Shannon divergence for the V3–V5 16S-based data. (TIFF) [file pcbi.1002863.s013.tiff]

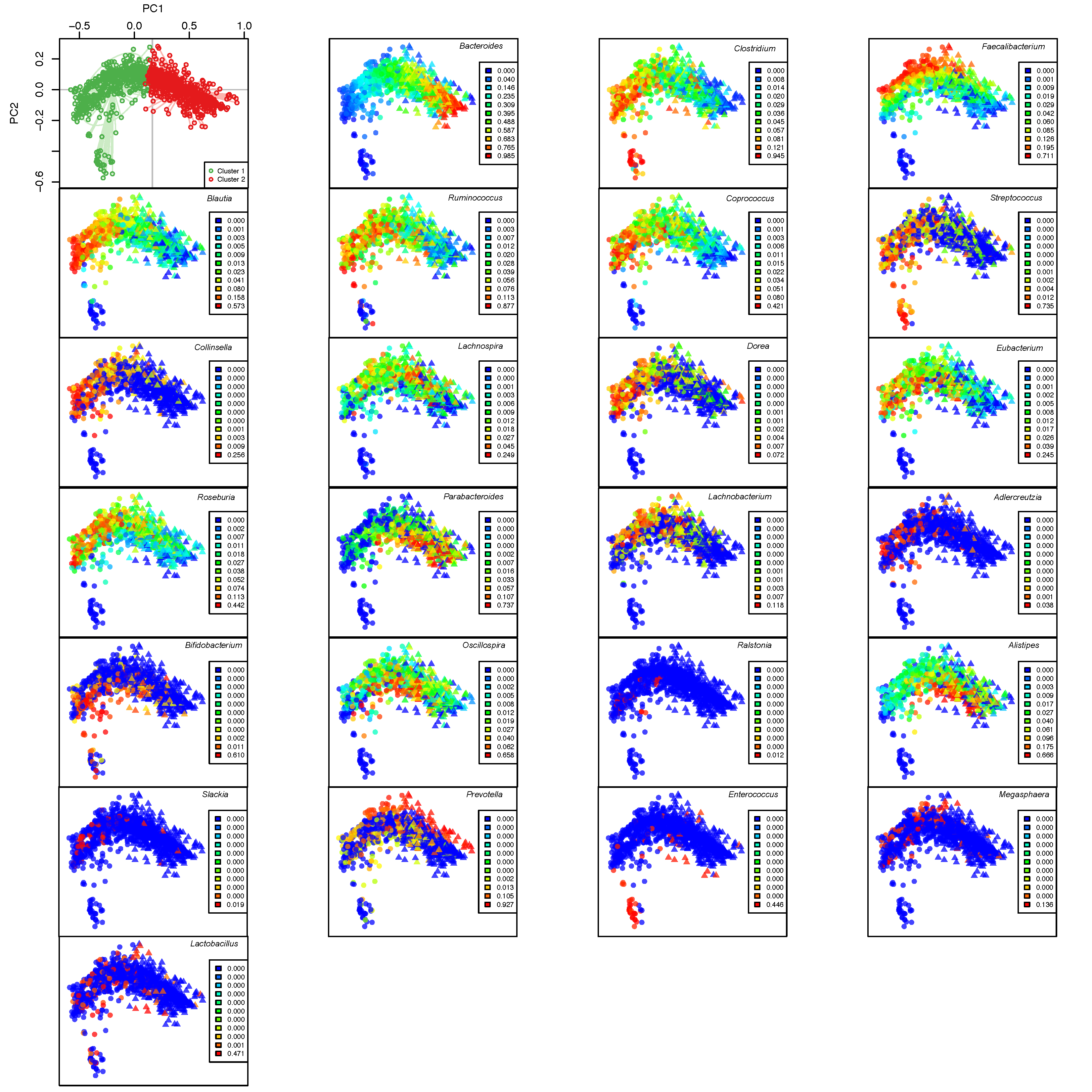

Supplement: Figure S14 — Gradients of the 24 taxa most highly correlated with the first 2 PCs in HMP fecal samples using weighted UniFrac for the V3–V5 16S-based data. (TIFF) [file pcbi.1002863.s014.tiff]

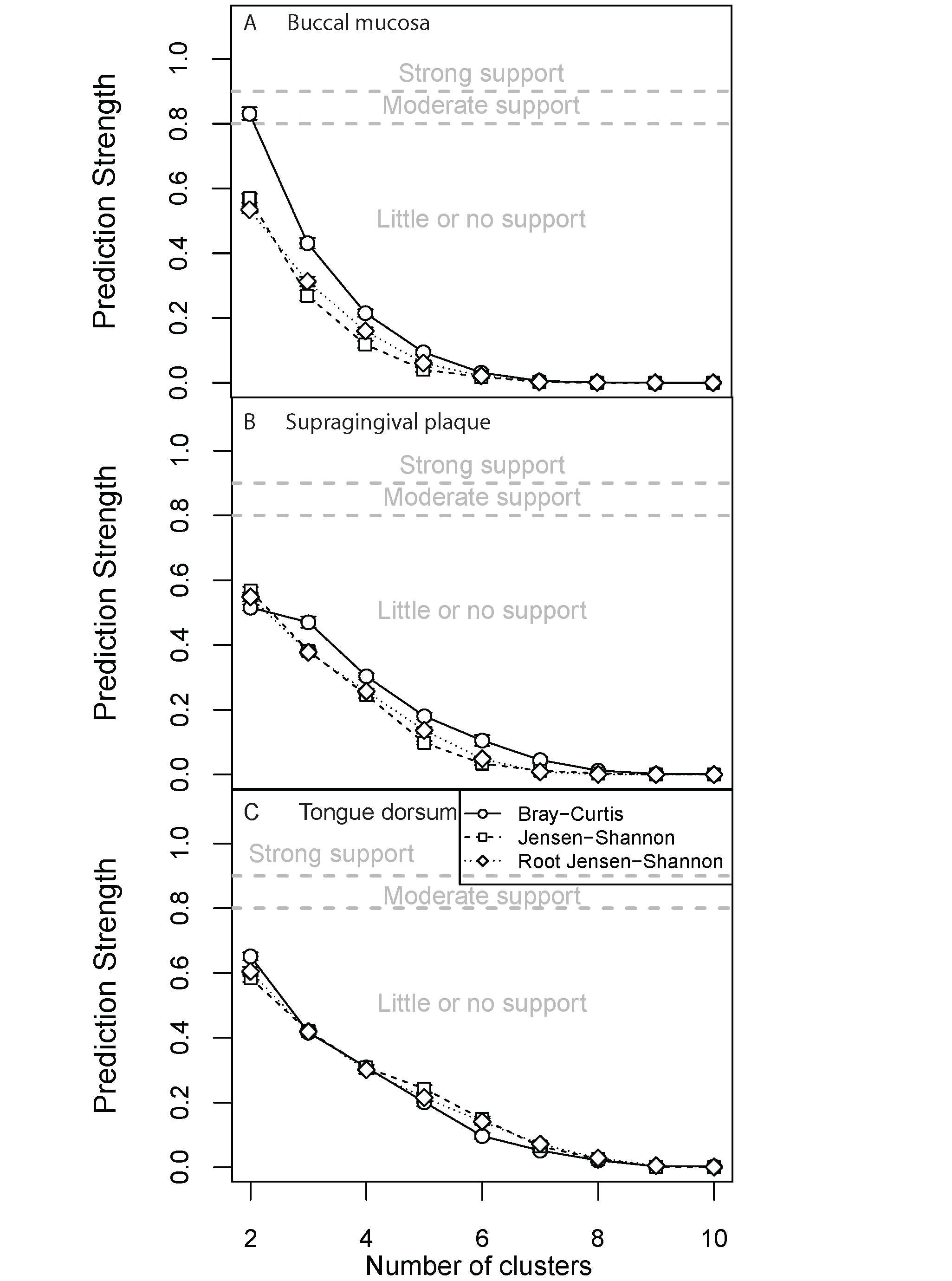

Supplement: Figure S15 — Prediction scores for enterotypes in HMP samples using WGS data. Prediction strength scores calculated using 3 distances metrics for buccal mucosa (A), supragingival (B), and tongue dorsum (C) samples. The thresholds for significance of clustering scores are indicated as dashed lines on the plots. Bars are standard errors. (TIFF) [file pcbi.1002863.s015.tiff]

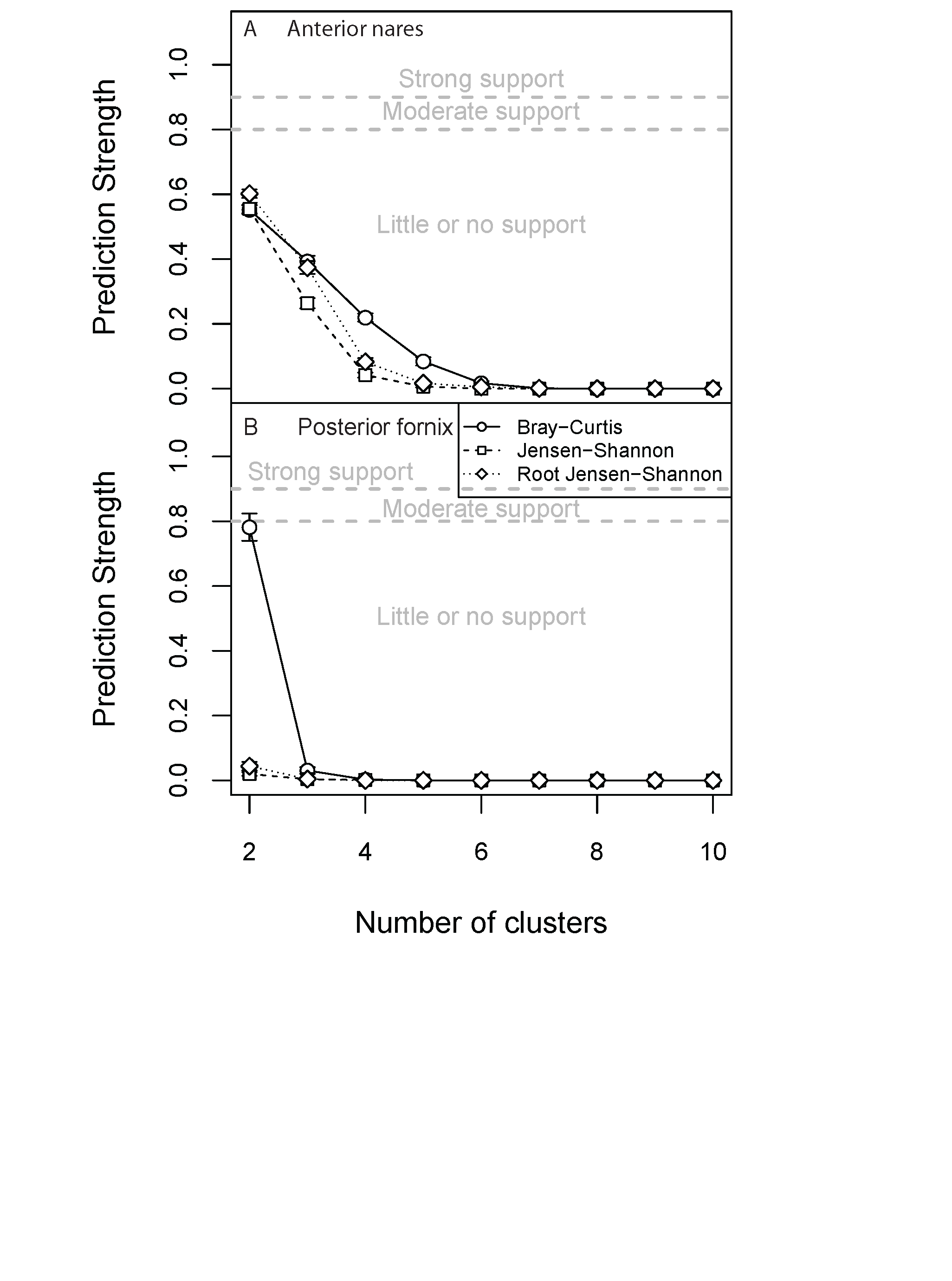

Supplement: Figure S16 — Prediction scores for enterotypes in HMP samples using WGS data. Prediction strength scores calculated using 3 distances metrics for anterior nares (A), and posterior fornix (B) samples. The thresholds for significance of clustering scores are indicated as dashed lines on the plots. Bars are standard errors. (TIFF) [file pcbi.1002863.s016.tiff]

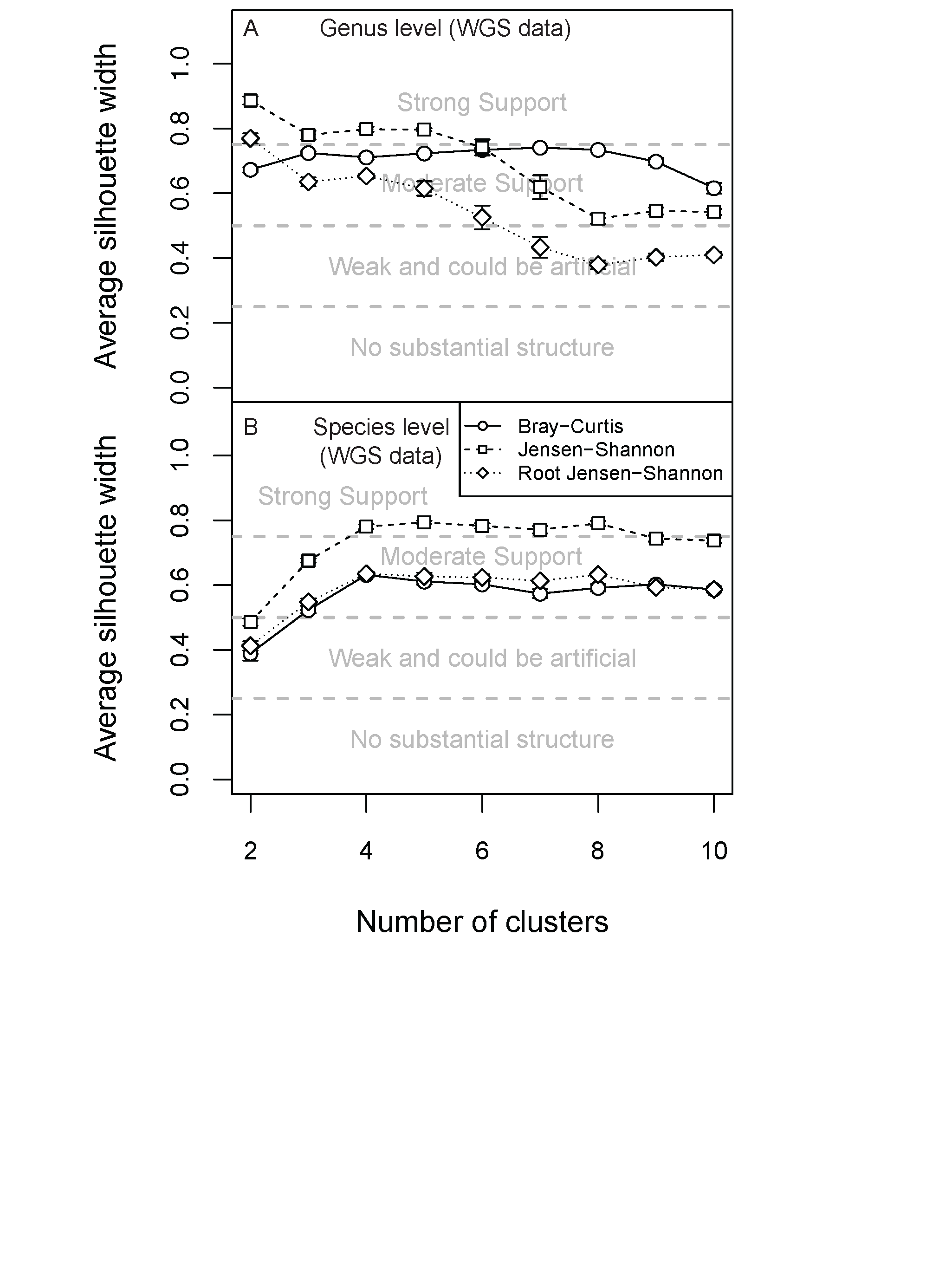

Supplement: Figure S17 — Silhouette index for enterotypes in HMP vaginal samples using WGS data at different taxonomic levels. Silhouette index scores calculated using 3 distances metrics for genus (A), and species (B) levels. The thresholds for significance of clustering scores are indicated as dashed lines on the plots. Bars are standard errors. (TIFF) [file pcbi.1002863.s017.tiff]

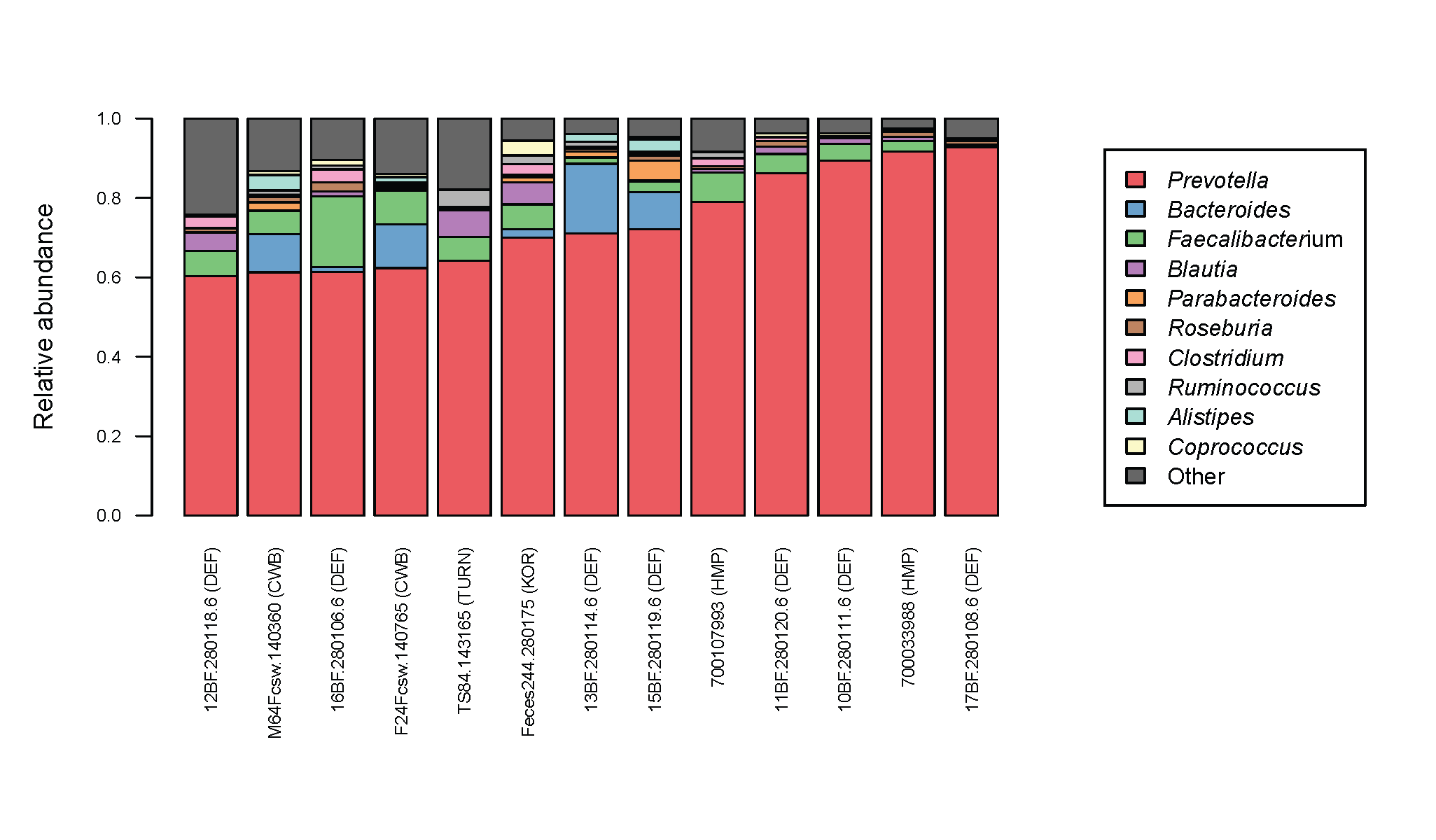

Supplement: Figure S18 — Taxon distribution for the 10 most common genera in the small number (13) of high Prevotella samples (≥0.6 relative abundance). (TIFF) [file pcbi.1002863.s018.tiff]

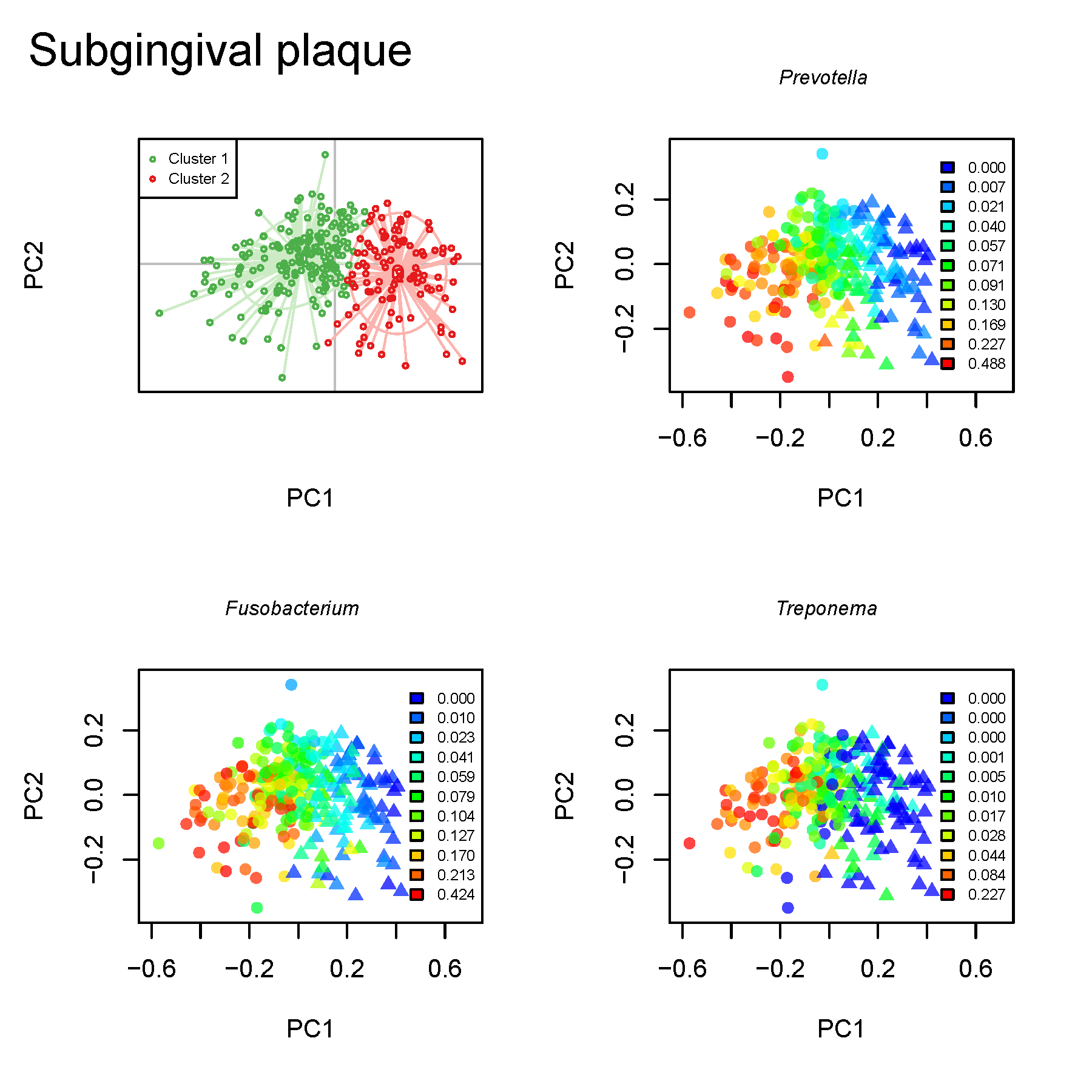

Supplement: Figure S19 — Gradients of Prevotella, Fusobacterium and Treponema abundances in subgingival plaque samples. HMP samples are shown in a principal coordinates analysis of unweighted UniFrac distances. Samples are colored according to (A) putative cluster membership and by their abundances (0–1, see legend inserts) of (B) Prevotella, (C) Fusobacterium and (D) Treponema. (TIFF) [file pcbi.1002863.s019.tiff]

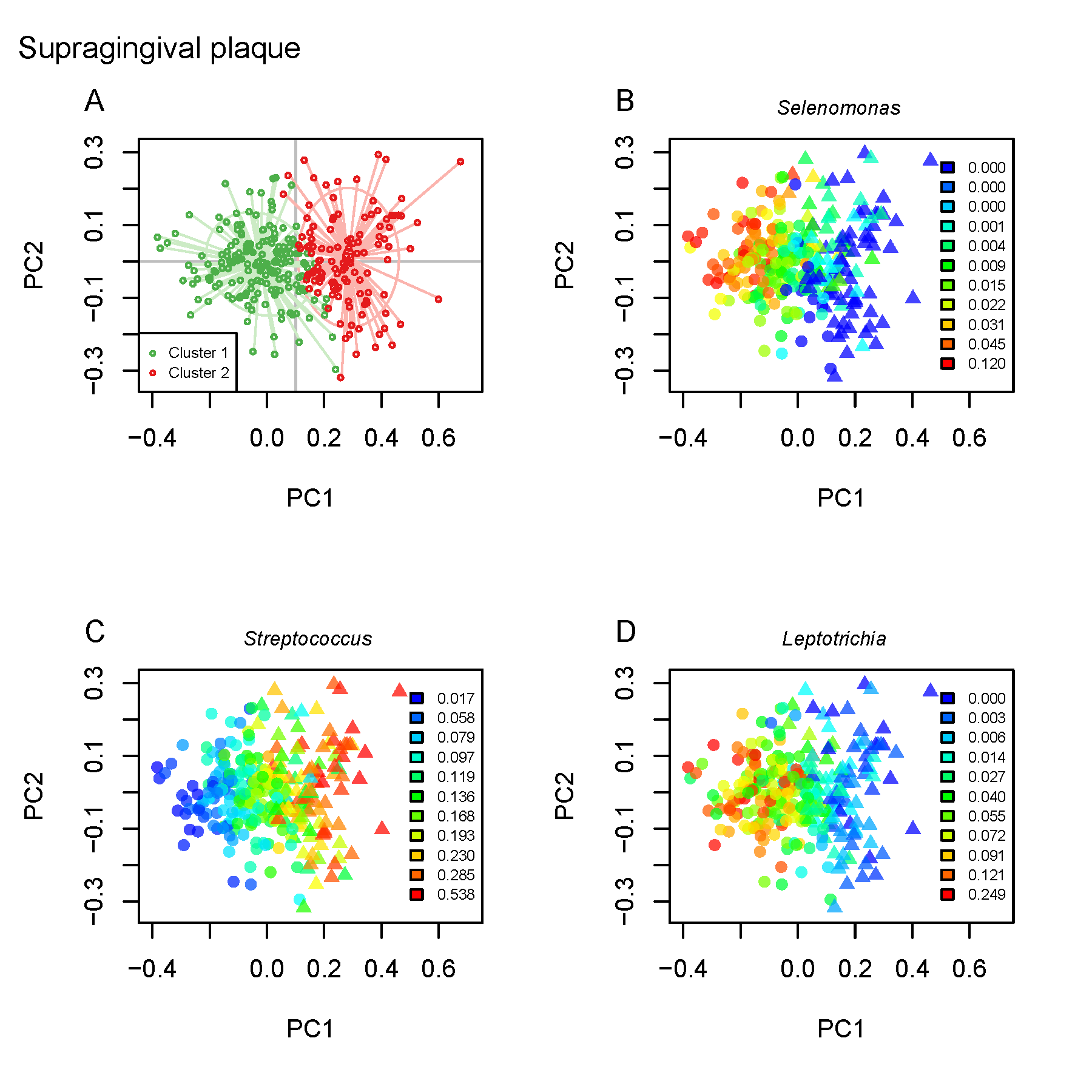

Supplement: Figure S20 — Gradients of Selenomonas, Streptococcus and Leptotrichia abundances in supragingival plaque samples. HMP samples are shown in a principal coordinates analysis of unweighted UniFrac distances. Samples are colored according to (A) putative cluster membership and by their abundances (0–1, see legend inserts) of (B) Selenomonas, (C) Streptococcus and (D) Leptotrichia. (TIFF) [file pcbi.1002863.s020.tiff]

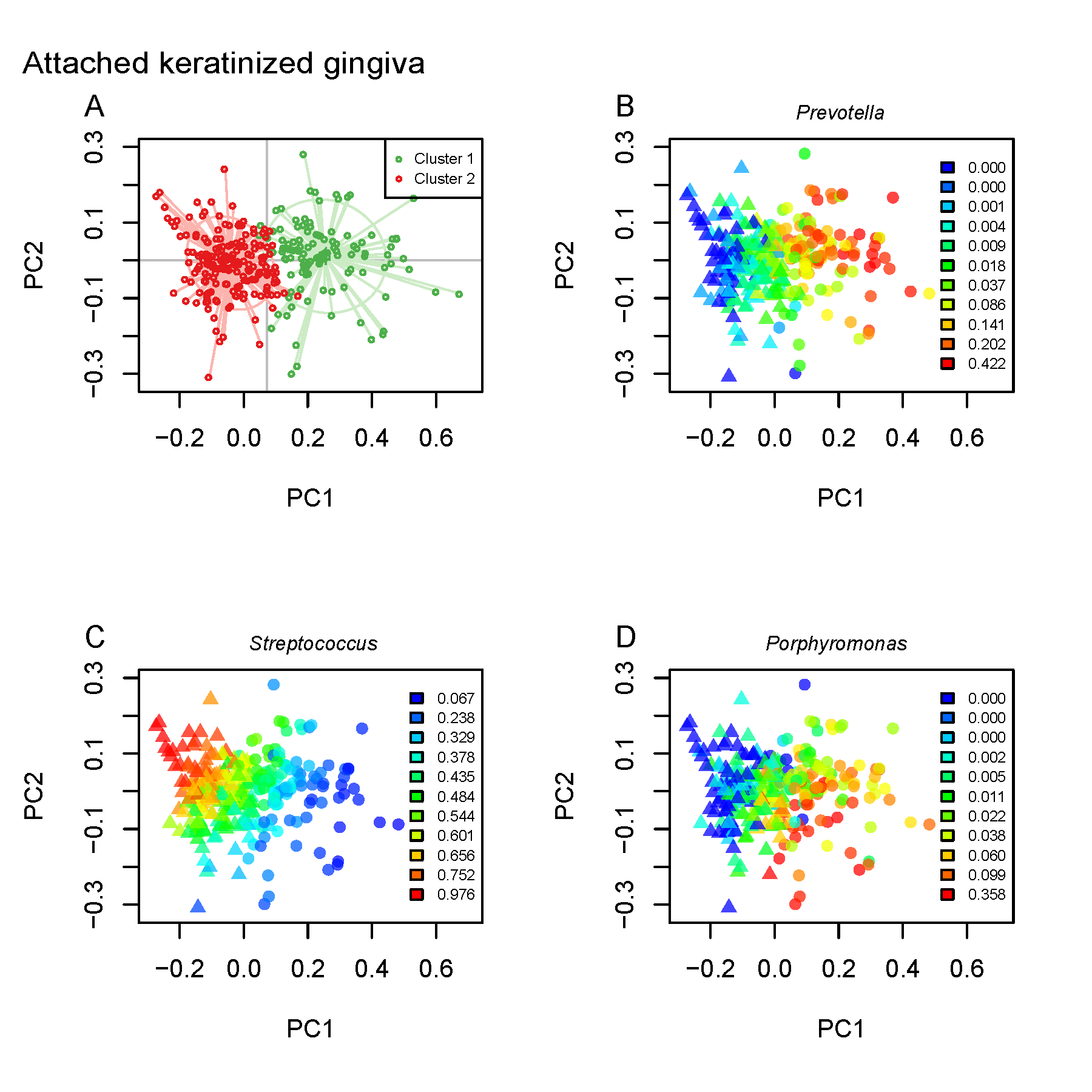

Supplement: Figure S21 — Gradients of Prevotella, Streptococcus and Porphyromonas abundances in attached keratinized gingiva samples. HMP samples are shown in a principal coordinates analysis of unweighted UniFrac distances. Samples are colored according to (A) putative cluster membership and by their abundances (0–1, see legend inserts) of (B) Prevotella, (C) Streptococcus and (D) Porphyromonas. (TIFF) [file pcbi.1002863.s021.tiff]

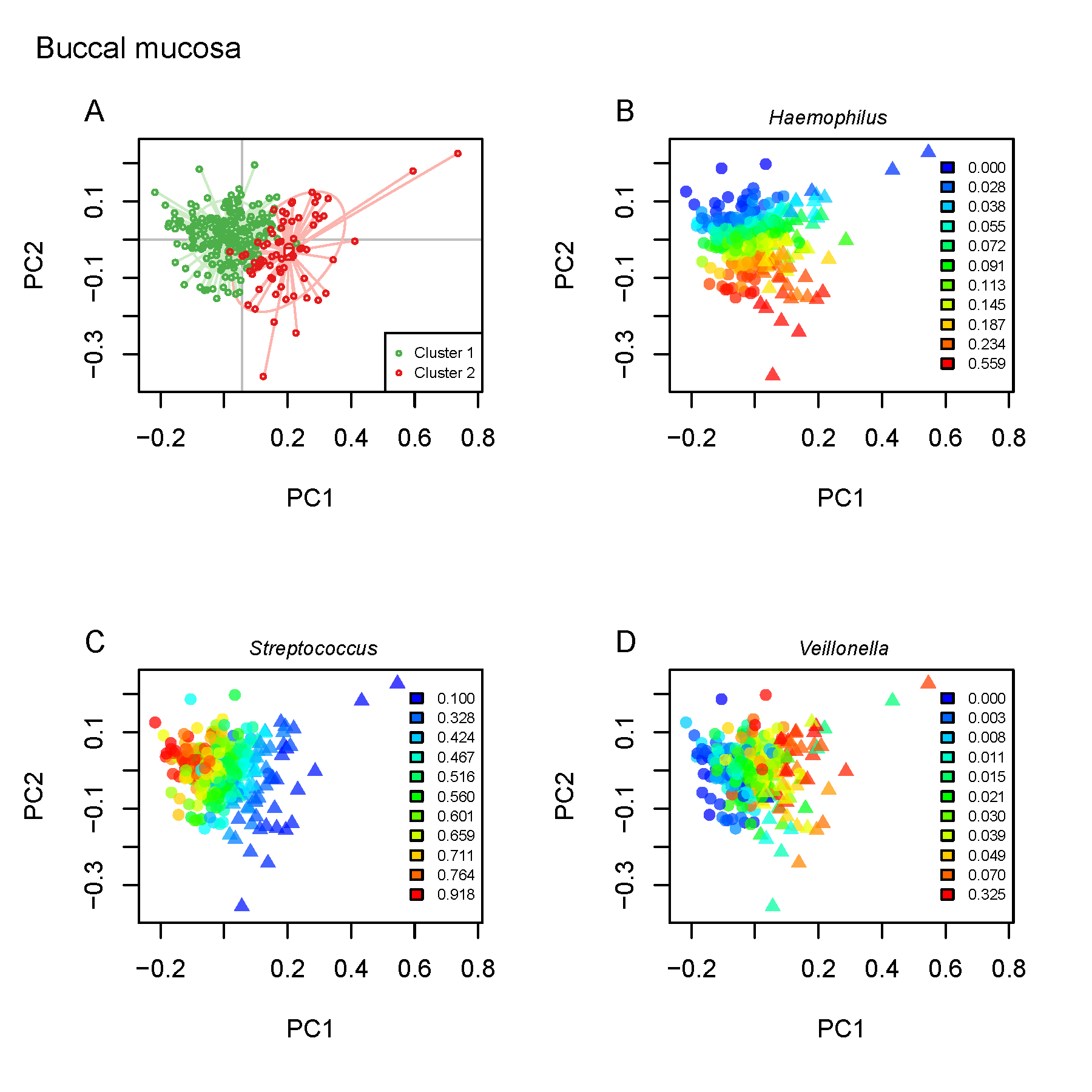

Supplement: Figure S22 — Gradients of Haemophilus, Streptococcus and Veillonella abundances in buccal mucosa samples. HMP samples are shown in a principal coordinates analysis of unweighted UniFrac distances. Samples are colored according to (A) putative cluster membership and by their abundances (0–1, see legend inserts) of (B) Haemophilus, (C) Streptococcus and (D) Veillonella. (TIFF) [file pcbi.1002863.s022.tiff]

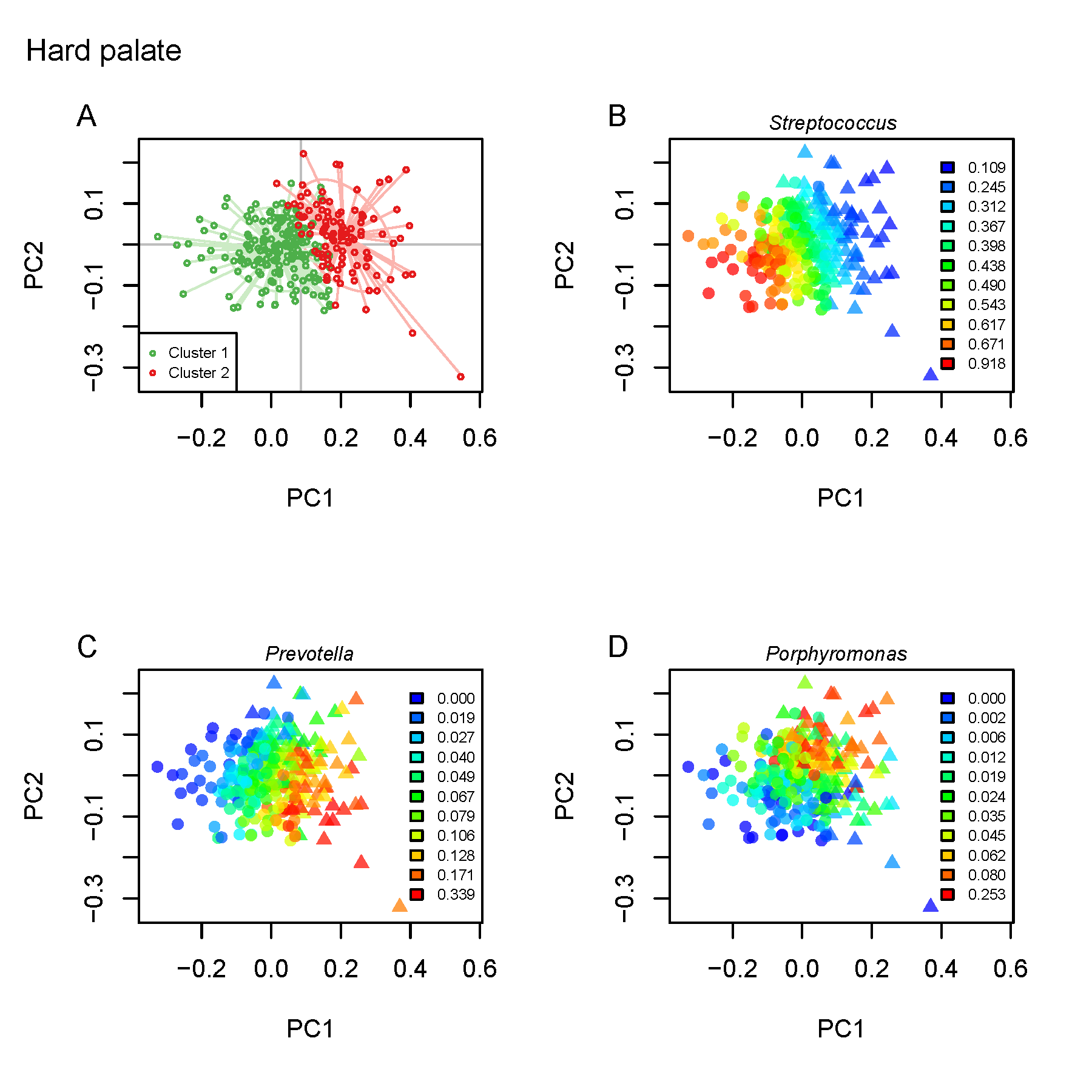

Supplement: Figure S23 — Gradients of Streptococcus, Prevotella and Porphyromonas abundances in hard palate samples. HMP samples are shown in a principal coordinates analysis of unweighted UniFrac distances. Samples are colored according to (A) putative cluster membership and by their abundances (0–1, see legend inserts) of (B) Streptococcus, (C) Prevotella and (D) Porphyromonas. (TIFF) [file pcbi.1002863.s023.tiff]

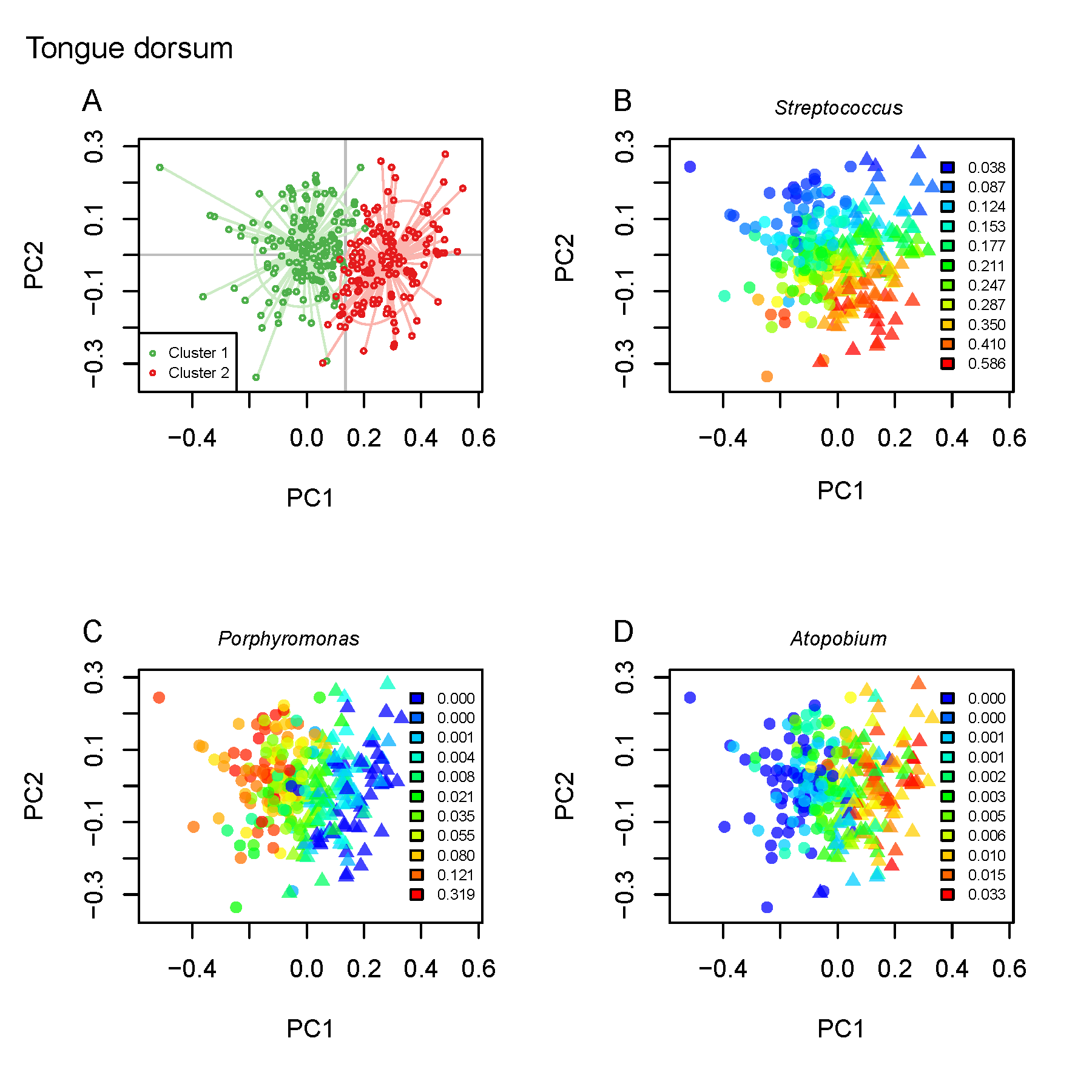

Supplement: Figure S24 — Gradients of Streptococcus, Porphyromonas and Atopobium abundances in tongue dorsum samples. HMP samples are shown in a principal coordinates analysis of unweighted UniFrac distances. Samples are colored according to (A) putative cluster membership and by their abundances (0–1, see legend inserts) of (B) Streptococcus, (C) Porphyromonas and (D) Atopobium. (TIFF) [file pcbi.1002863.s024.tiff]

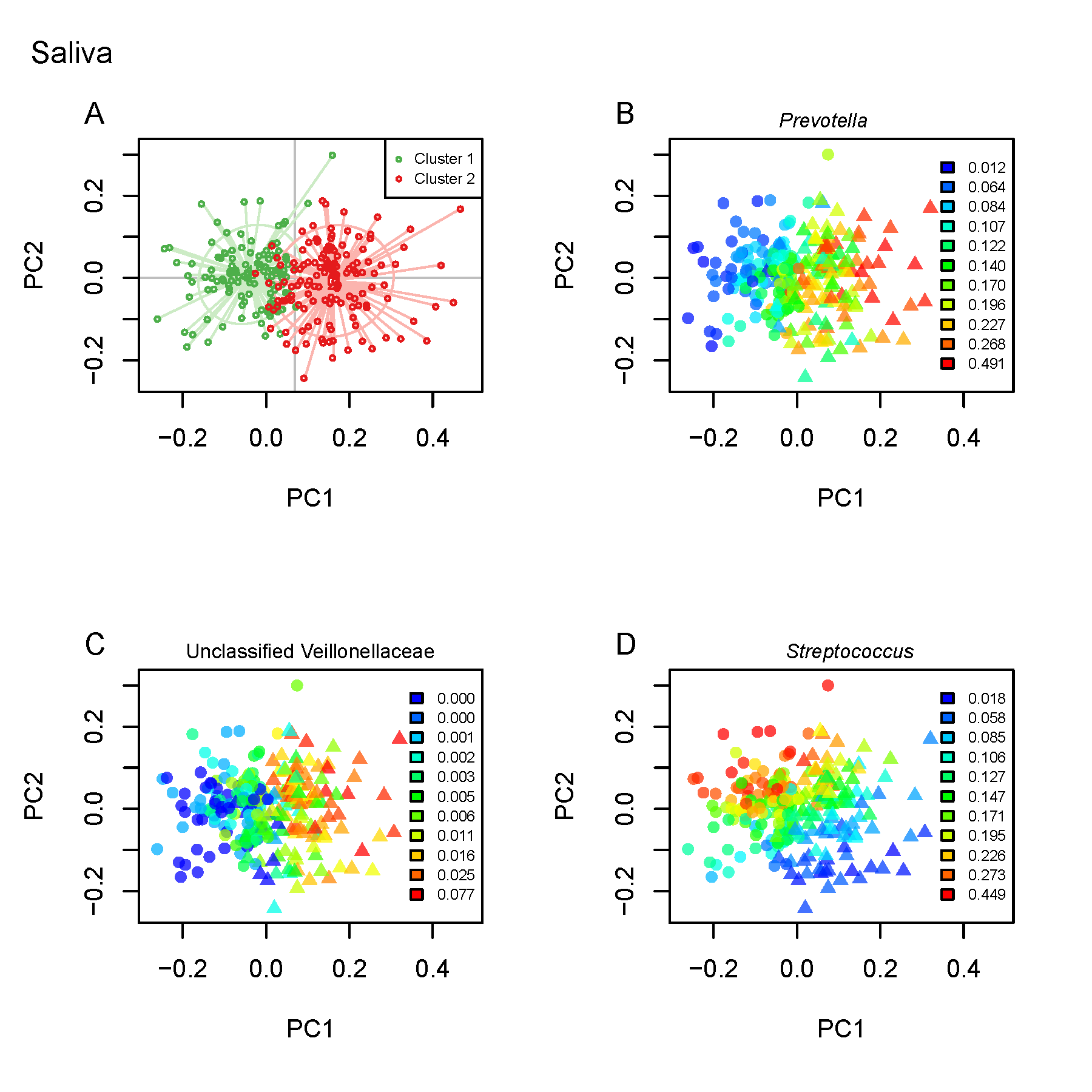

Supplement: Figure S25 — Gradients of Prevotella, Unclassified Veillonellaceae and Streptococcus abundances in saliva samples. HMP samples are shown in a principal coordinates analysis of unweighted UniFrac distances. Samples are colored according to (A) putative cluster membership and by their abundances (0–1, see legend inserts) of (B) Prevotella, (C) Unclassified Veillonellaceae and (D) Streptococcus. (TIFF) [file pcbi.1002863.s025.tiff]

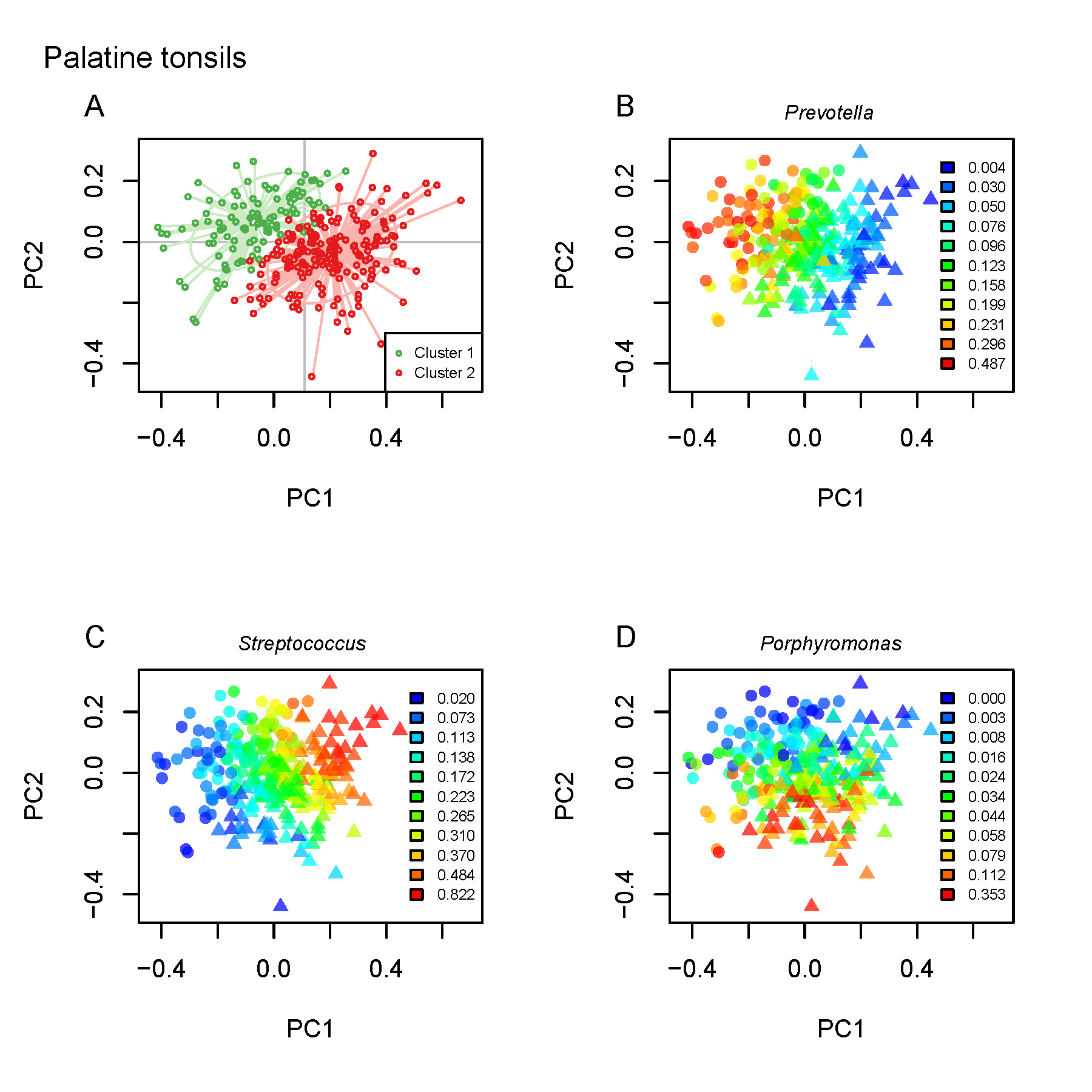

Supplement: Figure S26 — Gradients of Prevotella, Streptococcus and Porphyromonas abundances in palatine tonsils samples. HMP samples are shown in a principal coordinates analysis of unweighted UniFrac distances. Samples are colored according to their (A) putative cluster membership and by abundances (0–1, see legend inserts) of (B) Prevotella, (C) Streptococcus and (D) Porphyromonas. (TIFF) [file pcbi.1002863.s026.tiff]

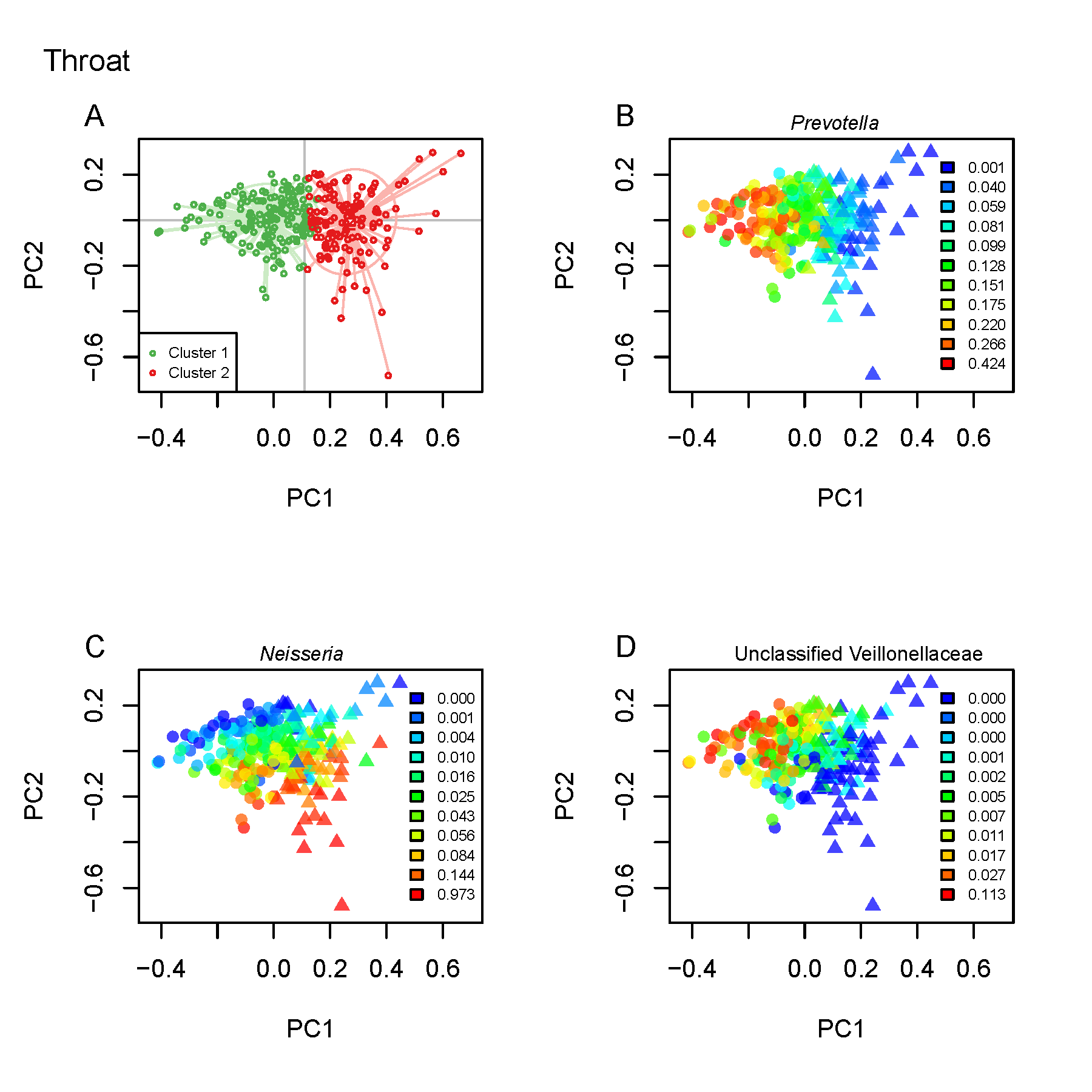

Supplement: Figure S27 — Gradients of Prevotella, Neisseria and Unclassified Veillonellaceae abundances in throat samples. HMP samples are shown in a principal coordinates analysis of unweighted UniFrac distances. Samples are colored according to (A) putative cluster membership and by their abundances (0–1, see legend inserts) of (B) Prevotella, (C) Neisseria and (D) Unclassified Veillonellaceae. (TIFF) [file pcbi.1002863.s027.tiff]

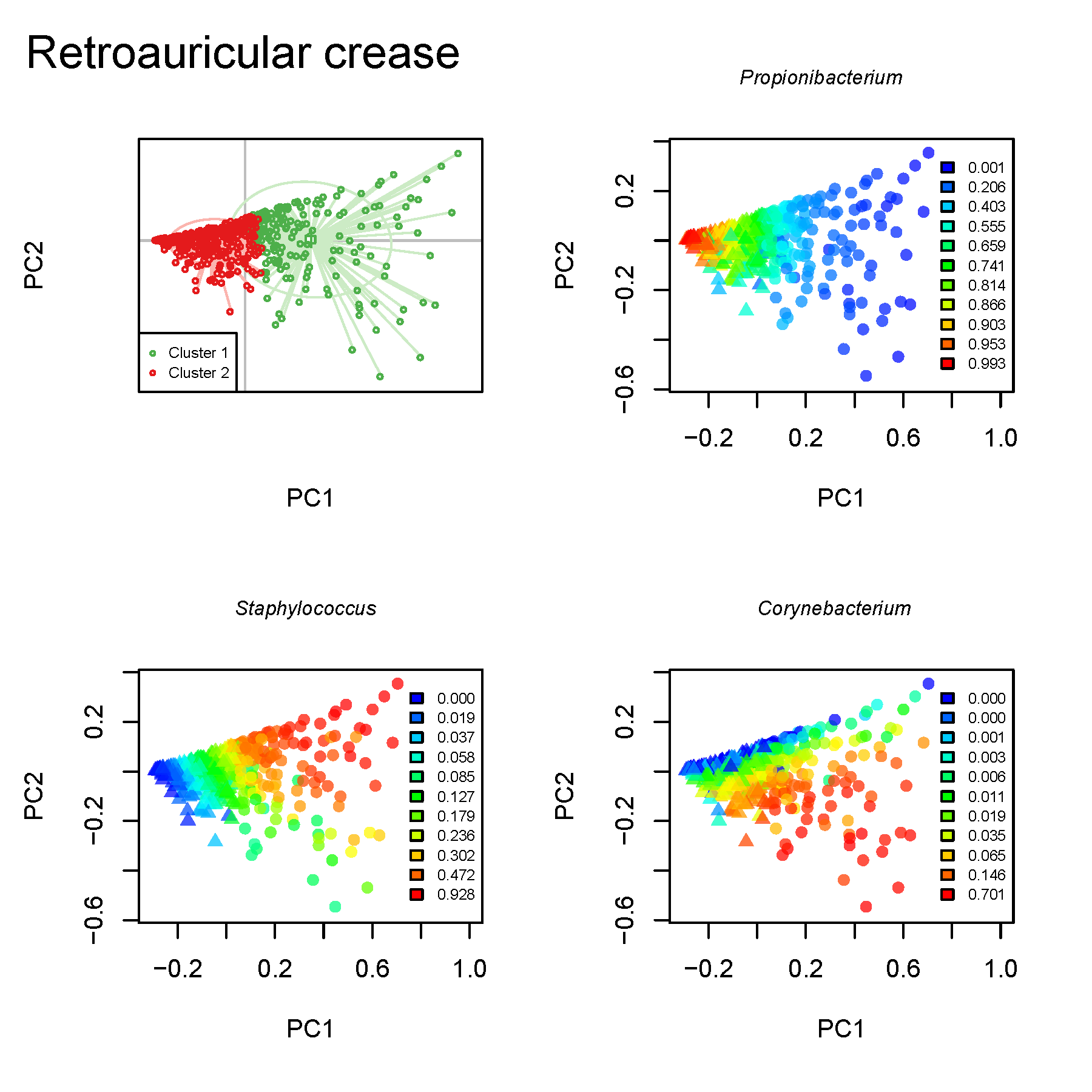

Supplement: Figure S28 — Gradients of Propionibacterium, Staphylococcus and Corynebacterium abundances in retroauricular crease samples. HMP samples are shown in a principal coordinates analysis of unweighted UniFrac distances. Samples are colored according to (A) putative cluster membership and by their abundances (0–1, see legend inserts) of (B) Propionibacterium, (C) Staphylococcus and (D) Corynebacterium. (TIFF) [file pcbi.1002863.s028.tiff]

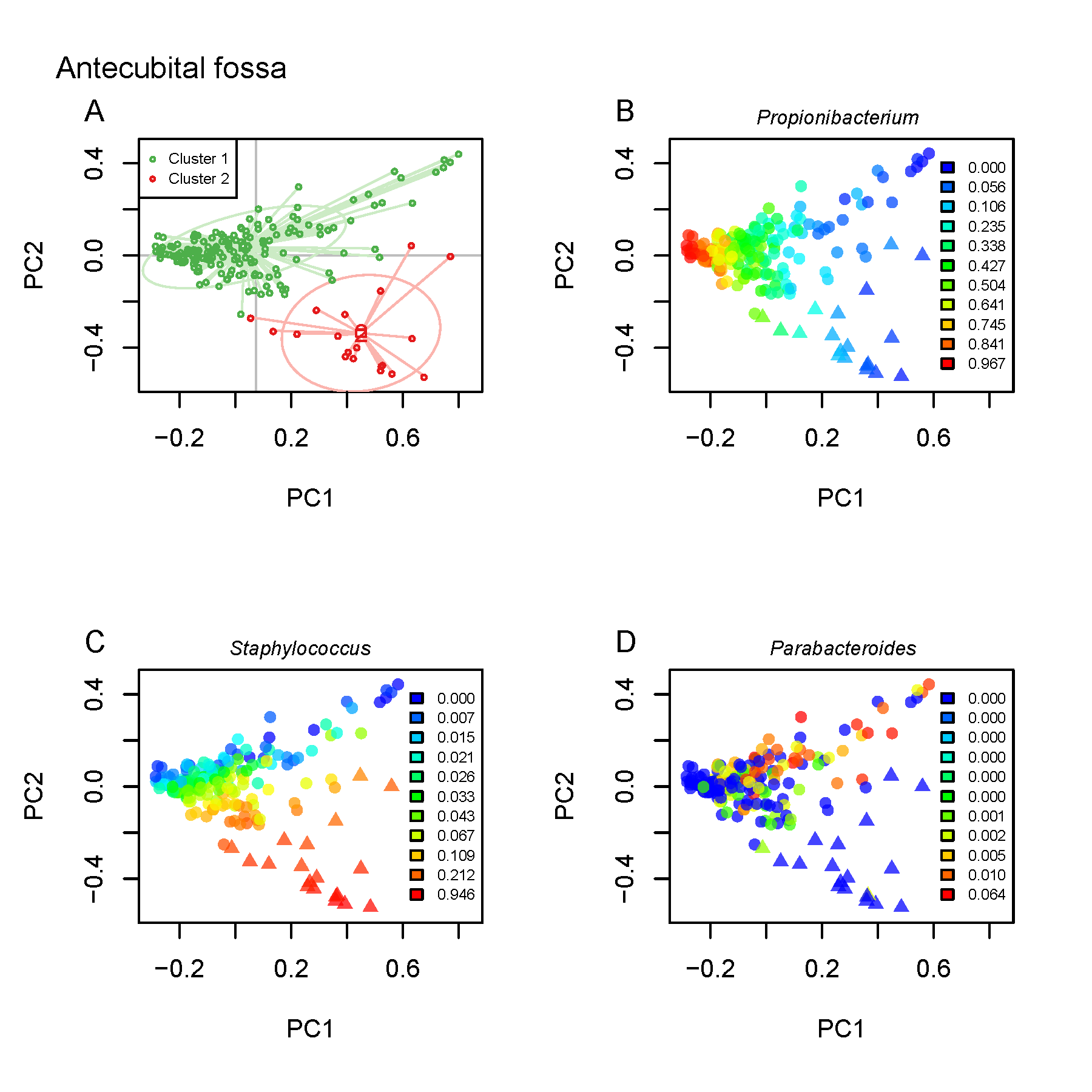

Supplement: Figure S29 — Gradients of Propionibacterium, Staphylococcus and Parabacteroides abundances in antecubital fossa samples. HMP samples are shown in a principal coordinates analysis of unweighted UniFrac distances. Samples are colored according to (A) putative cluster membership and by their abundances (0–1, see legend inserts) of (B) Propionibacterium, (C) Staphylococcus and (D) Parabacteroides. (TIFF) [file pcbi.1002863.s029.tiff]

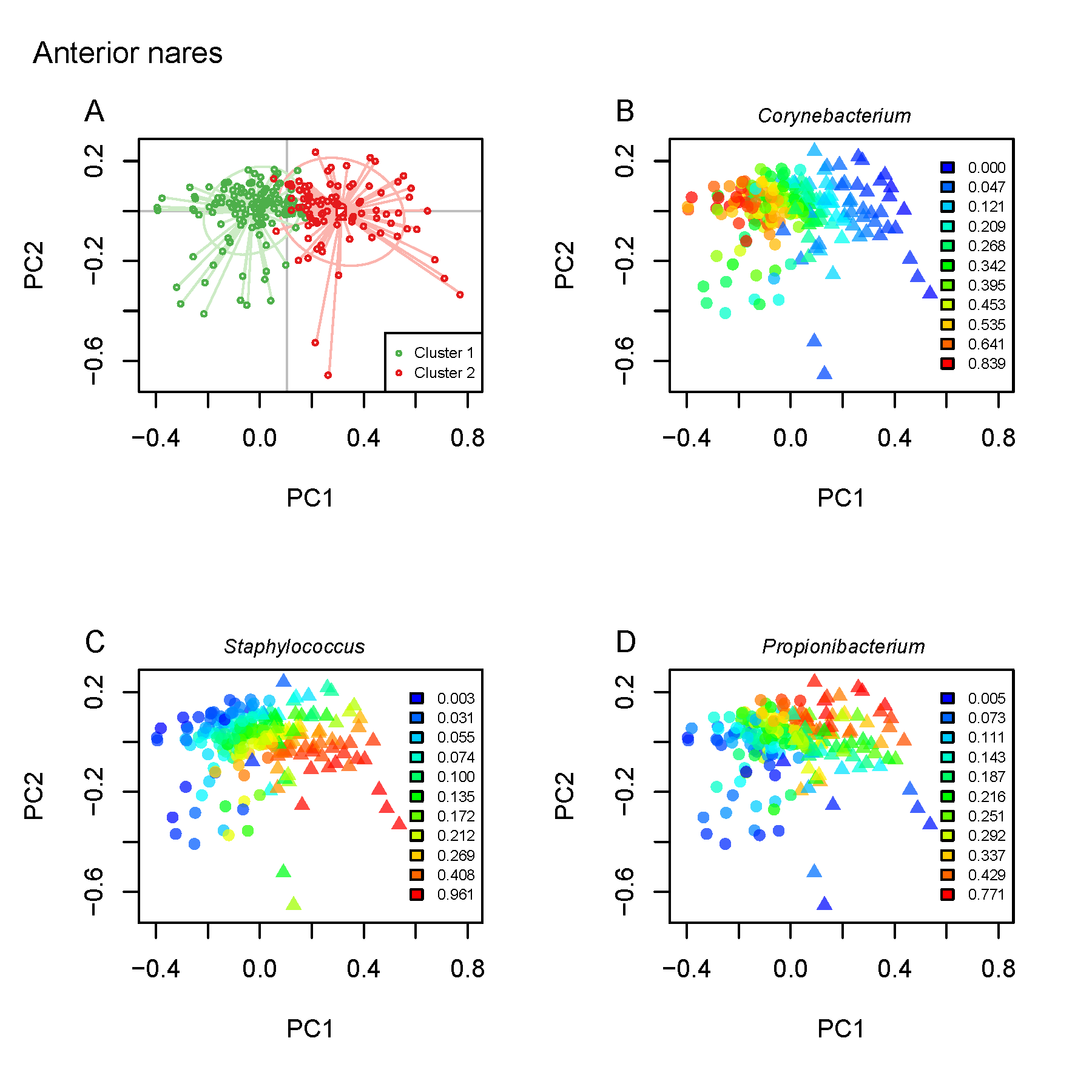

Supplement: Figure S30 — Gradients of Corynebacterium, Staphylococcus and Propionibacterium abundances in anterior nares samples. HMP samples are shown in a principal coordinates analysis of unweighted UniFrac distances. Samples are colored according to (A) putative cluster membership and by their abundances (0–1, see legend inserts) of (B) Corynebacterium, (C) Staphylococcus and (D) Propionibacterium. (TIFF) [file pcbi.1002863.s030.tiff]
