# Supplementary material for: A Guide to Enterotypes across the Human Body: Meta-Analysis of Microbial Community Structures in Human Microbiome Datasets
Source: PLoS Comput Biol. 2013 Jan 10;9(1):e1002863. doi: 10.1371/journal.pcbi.1002863 (PMC3542080; doi:10.1371/journal.pcbi.1002863)
Supplement: Table S1 — List of studies used in the data analysis. (DOCX) [file pcbi.1002863.s031.docx]

Table S1. List of studies used in the data analysis.

|  | **Study** | **Authors** | **Abbreviation** | **Age** | **Sex** | **Country** | **Sample Type** | **# of Samples** | **# of**  **Sequences** | **Reference** |
| --- | --- | --- | --- | --- | --- | --- | --- | --- | --- | --- |
| 1 | A core gut microbiome in obese and lean  twins | Turnbaugh *et al.* | Turn | 11.7±1.2  child | F | USA | Feces | 154 | 817,556 | [1] |
| 2 | Association between composition of the human gastrointestinal microbiome and development of fatty liver with choline deﬁciency | Spencer *et al.* | Fatty | 59±13.7  Adult | F | USA | Feces | 74 | 173,215 | [2] |
| 3 | Bacterial community variation in human body habitats across space and time | Costello *et al*. | CWB | Adult  (30-35, 60) | M+F | USA | Feces, Skin, Oral, External auditory canal, Nostril and Hair | 815 | 699,431 | [3] |
| 4 | Characterization of bacteria in biopsies of colon and stools by high throughput sequencing of the V2 region of bacterial 16S rRNA gene in human | Momozawa *et al.* | MOMO | Adult  (25-62) | M+F | Europe | Cecum, Ascending colon, Transverse colon, Sigmoid colon, Colonoscopy stool, and Feces | 70 | 63,999 | [4] |
| 5 | Composition, variability, and temporal stability of the intestinal microbiota of the elderly | Claesson *et al.* | Eld | Elderly  (78±7) | M+F | Europe (Ireland) | Feces | 168 | 2,400,407 | [5] |
| 6 | Delivery mode shapes the acquisition and structure of the initial microbiota across multiple body habitats in newborns | Dominguez-Bello *et al.* | DOM | Adults  (21-33)  Infants | F | Venezuela | Skin, Oral and Vagina | 187 | 145,055 | [6] |
| 7 | Disordered microbial communities in the upper respiratory tract of cigarette smokers | Charlson *et al.* | SMOKE | Adults  (20-61) | M+F | USA | Nose and Throat | 291 | 284,535 | [7] |
| 8 | Forensic identification using skin bacterial communities | Fierer *et al.* | FFOR | Adults  (20-35) | M+F | USA | Skin | 40 | 44,848 | [8] |
| 9 | Human oral, gut, and plaque microbiota in patients with atherosclerosis | Koren *et al.* | Kor | Adult  68 | M+F | Europe  (Sweden) | Feces, Oral, Atherosclerotic plaques | 73 | 320,494 | [9] |
| 10 | Impact of diet in shaping gut microbiota revealed by a comparative study in children from Europe and rural Africa | De Filippo *et al.* | Def | Children  (1-6) | M+F | Africa  (Burkina Faso)  Europe  (Italy) | Feces | 29 | 74,032 | [10] |
| 11 | Incomplete recovery and individualized responses of the human distal gut microbiota to repeated antibiotic perturbation | Dethlefsen and Relman | Deth | Adults  (28-54) | F | USA | Feces | 198 | 3,087,840 | [11] |
| 12 | Resistant starches types 2 and 4 have differential effects on the composition of the fecal microbiota in human subjects | Martinez *et al.* | STAR | Adults  (28-38) | M+F | USA | Feces | 162 | 180,341 | [12] |
| 13 | Short-term antibiotic treatment has differing long-term impacts on the human throat and gut microbiome | Jakobsson *et al.* | ABX | Adults  (66±9.3) | M+F | Europe  (Sweden) | Feces and throat | 58 | 95,491 | [13] |
| 14 | Succession of microbial consortia in the developing infant gut microbiome | Koenig *et al.* | Koen | Infant | M | USA | Feces | 62 | 272,327 | [14] |
| 15 | The influence of sex, handedness, and washing on the diversity of hand surface bacteria | Fierer *et al.* | FUND | Adults | M+F | USA | Skin | 102 | 336,212 | [15] |
| 16 | Vaginal microbiome of reproductive-age women | Ravel *et al.* | UMIGS | Adults  (30.6±7.34) | F | USA | Vagina | 394 | 796,280 | [16] |
| 17 | Metahit | Metahit | MH | Adults | M+F | Denmark | Feces | 85 | 3,977,682,656 | [17] |
| 18 | Human Microbiome Project 16S rRNA data | The human Microbiome Project Consortium | HMP | Adults  (27±5) | M+F | USA | Feces, Vagina, Skin, Oral, Airway | 10,213 | 20,472,845 | [18] |
| 19 | Human Microbiome Project WGS data | The human Microbiome Project Consortium | HMP | Adults  (27±5) | M+F | USA | Feces, Vagina, Skin, Oral, Airway | 690 | > 35,000,000,000 | [18] |

1. Turnbaugh PJ, Hamady M, Yatsunenko T, Cantarel BL, Duncan A, et al. (2009) A core gut microbiome in obese and lean twins. Nature 457: 480-484.

2. Spencer MD, Hamp TJ, Reid RW, Fischer LM, Zeisel SH, et al. (2011) Association between composition of the human gastrointestinal microbiome and development of fatty liver with choline deficiency. Gastroenterology 140: 976-986.

3. Costello EK, Lauber CL, Hamady M, Fierer N, Gordon JI, et al. (2009) Bacterial community variation in human body habitats across space and time. Science 326: 1694-1697.

4. Momozawa Y, Deffontaine V, Louis E, Medrano JF (2011) Characterization of bacteria in biopsies of colon and stools by high throughput sequencing of the V2 region of bacterial 16S rRNA gene in human. PLoS ONE 6: e16952.

5. Claesson MJ, Cusack S, O'Sullivan O, Greene-Diniz R, de Weerd H, et al. (2011) Composition, variability, and temporal stability of the intestinal microbiota of the elderly. Proc Natl Acad Sci U S A 108 Suppl 1: 4586-4591.

6. Dominguez-Bello MG, Costello EK, Contreras M, Magris M, Hidalgo G, et al. (2010) Delivery mode shapes the acquisition and structure of the initial microbiota across multiple body habitats in newborns. Proc Natl Acad Sci U S A 107: 11971-11975.

7. Charlson ES, Chen J, Custers-Allen R, Bittinger K, Li H, et al. (2010) Disordered microbial communities in the upper respiratory tract of cigarette smokers. PLoS ONE 5: e15216.

8. Fierer N, Lauber CL, Zhou N, McDonald D, Costello EK, et al. (2010) Forensic identification using skin bacterial communities. Proc Natl Acad Sci U S A 107: 6477-6481.

9. Koren O, Spor A, Felin J, Fak F, Stombaugh J, et al. (2011) Human oral, gut, and plaque microbiota in patients with atherosclerosis. Proc Natl Acad Sci U S A 108 Suppl 1: 4592-4598.

10. De Filippo C, Cavalieri D, Di Paola M, Ramazzotti M, Poullet JB, et al. (2010) Impact of diet in shaping gut microbiota revealed by a comparative study in children from Europe and rural Africa. Proc Natl Acad Sci U S A 107: 14691-14696.

11. Dethlefsen L, Relman DA (2011) Incomplete recovery and individualized responses of the human distal gut microbiota to repeated antibiotic perturbation. Proc Natl Acad Sci U S A 108 Suppl 1: 4554-4561.

12. Martinez I, Kim J, Duffy PR, Schlegel VL, Walter J (2010) Resistant starches types 2 and 4 have differential effects on the composition of the fecal microbiota in human subjects. PLoS ONE 5: e15046.

13. Jakobsson HE, Jernberg C, Andersson AF, Sjolund-Karlsson M, Jansson JK, et al. (2010) Short-term antibiotic treatment has differing long-term impacts on the human throat and gut microbiome. PLoS One 5: e9836.

14. Koenig JE, Spor A, Scalfone N, Fricker AD, Stombaugh J, et al. (2011) Succession of microbial consortia in the developing infant gut microbiome. Proc Natl Acad Sci U S A 108 Suppl 1: 4578-4585.

15. Fierer N, Hamady M, Lauber CL, Knight R (2008) The influence of sex, handedness, and washing on the diversity of hand surface bacteria. Proc Natl Acad Sci U S A 105: 17994-17999.

16. Ravel J, Gajer P, Abdo Z, Schneider GM, Koenig SS, et al. (2011) Vaginal microbiome of reproductive-age women. Proc Natl Acad Sci U S A 108 Suppl 1: 4680-4687.

17. Arumugam M, Raes J, Pelletier E, Le Paslier D, Yamada T, et al. (2011) Enterotypes of the human gut microbiome. Nature 473: 174-180.

18. The Human Microbiome Project Consortium (2012) Structure, function and diversity of the healthy human microbiome. Nature 486: 207-214.
